# ‘Paper care not patient care’: Nurse and patient experiences of comprehensive risk assessment and care plan documentation in hospital

**DOI:** 10.1111/jocn.16291

**Published:** 2022-03-29

**Authors:** Catherine Paterson, Cara Roberts, Kasia Bail

**Affiliations:** ^1^ 2234 School of Nursing, Midwifery and Public Health University of Canberra Bruce Australian Capital Territory Australia; ^2^ Canberra Health Services and ACT Health SYNERGY Nursing and Midwifery Research Centre Canberra Hospital Garran Australian Capital Territory Australia; ^3^ School of Nursing, Midwifery and Paramedicine Robert Gordon University Aberdeen UK

**Keywords:** care plan, documentation, multidisciplinary team, nurses, patients, preventable harms, qualitative study, risk assessment

## Abstract

**Aims and Objectives:**

To explore organisation‐wide experiences of person‐centred care and risk assessment practices using existing healthcare organisation documentation.

**Background:**

There is increasing emphasis on multidimensional risk assessments during hospital admission. However, little is known about how nurses use multidimensional assessment documentation in clinical practice to address preventable harms and optimise person‐centred care.

**Design:**

A qualitative descriptive study reported according to COREQ.

**Methods:**

Metropolitan tertiary hospital and rehabilitation hospital servicing a population of 550,000. A sample of 111 participants (12 patients, 4 family members/carers, 94 nurses and 1 allied health professional) from a range of wards/clinical locations. Semi‐structured interviews and focus groups were conducted at two time points. The audio recording was transcribed, and an inductive thematic analysis was used to provide insight from multiple perspectives.

**Results:**

Three main themes emerged: (1) ‘What works well in practice’ included: efficiency in the structure of the documentation; the Introduction, Situation, Background Assessment, Recommendation (ISBAR) framework and prompting for clinical decision‐making were valued by nurses; and direct patient care is always prioritised. (2) ‘What does not work well in practice’: obtaining the patient's signature on daily care plans; multidisciplinary (MDT) involvement; duplication of paperwork and person‐centred goals are not well‐captured in care plan documentation. (3) ‘Experience of care’; satisfaction of person‐centred care; communication in the MDT was important, but sometimes insufficient; patients had variable involvement in their daily care plan; and inadequate integration of care between MDT team which negatively impacted patients.

**Conclusions:**

Efficient and streamlined documentation systems should herald feedback from nurses to address their clinical workflow needs and can support, and capture, their decision‐making that enables partnership with patients to improve the individualisation of care provision.

**Relevance to clinical practice:**

The integration of effective MDT involvement in clinical documentation was problematic and resulted in unmet supportive care from the patient's perspective.

**What does this paper contribute to the wider global clinical community?**

- Nurses prioritise direct patient care over documentation requirements, but efficient documentation structures are valued by nurses when they can quickly inform their patient focused care.
- Nurses and patients experience a lack of integration with multidisciplinary teams, which is reinforced by documentation structures.
- Nurses value text boxes which enable them to document the clinical nurse decision‐making they conduct in the process of individualising care.

## 1 INTRODUCTION

Preventable harms in hospital are defined as presence of an identifiable, modifiable cause of harm (Nabhan et al., 2012). Preventable harms are an unpleasant and even deadly experience for patients and their families, and a significant burden on the healthcare system (Bail et al., 2015; Berry et al., 2020; Thornton et al., 2017). These unnecessary harms in healthcare result in substantial economic cost (Slawomirski et al., 2017), morbidity and mortality as a result from suboptimal quality health care (Panagioti et al., 2019). Preventable harms are associated with nursing care and include pressure injury, infection, detrimental nutrition and hydration, falls, delirium, self‐harm or suicide, aggression and cognitive and functional decline (Australian Commission on Safety & Quality in Health Care, 2018a). The World Health Organisation defines patient harm as an incident that results in harm to a patient such as impairment of structure or function of the body and/or any deleterious effect arising or associated with plans or actions taken during the provision of health care, rather than an underlying disease or injury, and may be physical, social or psychological (e.g. disease, injury, suffering, disability and death) (World Alliance For Patient Safety Drafting Group et al., 2009). The challenge of preventing harm is complex in the real‐world setting because of interdependent risks and existing complex comorbidities, and complicated settings that include multiple healthcare professionals and organisational factors (Mallidou et al., 2011). Evidence has identified that 17% of healthcare expenditure is consumed by the direct sequelae of health care‐related patient harm (Jackson et al., 2011). The issue of patient safety is also intertwined with effective communication, particularly in inter‐ and intra‐hospital transfers. Improving patient safety can be achieved through structured tools such as the ISBAR technique, I: corresponds to the Identification, S: Current Situation, B: Background, A: Assessment and R: Recommendations. The ISBAR is used to standardise communication to promote patient safety in situations of transitions of care (Figueiredo & Potra, 2019).

## 2 BACKGROUND

Globally, there is recognition that health providers should undertake a comprehensive assessment of patient safety risks, which are often managed through organisational clinical documentation (Simsekler et al., 2019). The benefits of a comprehensive multidimensional assessment are to detect risks and identify care needs to inform interventions and plans of care to improve patient outcomes (Ellis et al., 2017). However, most assessment and screening include duplication of items and a high burden on nursing staff (Redley & Raggatt, 2017). Little is known about how healthcare professionals use multidimensional assessment documentation in clinical practice to address preventable harms and optimise person‐centred care in day‐to‐day practice.

Person‐centred care is the hallmark and touchstone of nursing practice (McCormack & McCance, 2006) and theory (Byrne et al., 2020). There is continual growing focus on the promotion and advocacy of person‐centred, individualised care, which is embedded in the healthcare discourse associated with safety and quality in health services (Australian Commission on Safety and Quality in Healthcare, 2018b; Sharp et al., 2018; World Health Organization, 2018). Therefore, healthcare organisations are developing clinical documentation that aim to screen for preventable harms and simultaneously support person‐centred care (Feo & Kitson, 2016; Harper et al., 2020; Rossiter et al., 2020).

To date, this is largest qualitative prospective study which set out to explore organisation wide experiences of person‐centred care and risk assessment practices using existing healthcare organisation documentation (Muinga et al., 2021; Saranto & Kinnunen, 2009). It is widely acknowledged that care documentation serves to support administrative processes that nurses perform, forms the legal document of care provided and creates a record of care that can be used for quality improvement, research and education (Australian Commission on Safety and Quality in Health Care, 2018a). Nursing documentation serves several important functions, and good nursing care depends crucially on access to high quality information. Documentation within clinical care facilities should provide information flow between multidisciplinary (MDT) healthcare providers (Brown et al., 2021), supports continuity of care for patients (Morey et al., 2021) and supports the clinician's memory of care provided.

The aim of this study was to explore experiences of person‐centred care and risk assessment practices using existing organisational healthcare documentation from the perspectives of healthcare professionals and patients.

## 3 METHODS

### 3.1 Design

A qualitative descriptive study (Sandelowski, 2000) was chosen to gain insight into healthcare professional and patient experiences of clinical documentation practice across different clinical specialties. Qualitative descriptive design was considered the most appropriate for an in‐depth examination, through semi‐structured individual interviews of patients' experiences (Kallio et al., 2016) and focus groups (Kitzinger, 1995) with nurses, allied health and medical professionals. The project has been reported according to the consolidated criteria for reporting qualitative studies (COREQ) 32‐item checklist, see Table S1 for completed checklist (Booth et al., 2014).

### 3.2 Setting

The setting is metropolitan tertiary acute hospital with 600 beds complemented by a 140‐bed rehabilitation hospital, serving a population of about 550,000. The clinical areas represented in this study included the following divisions: Surgical, Medical, Rehabilitation Aged and Community, Cancer and Ambulatory Support, Critical Care, Antenatal and Gynecological and Mental Health, Justice Health and Alcohol and Drug services. Eight staff focus groups and five patient interviews were conducted at Time 1 during May 2020 to explore experiences of using the Patient Care and Accountability Care Plan (PCAP) documentation in practice, see Supplementary File 1. Seven staff focus groups and eleven patient interviews were carried out at Time 2 in the same divisions during July and August 2020 to explore experiences of using the new pilot Integrated Risk Screening and Comprehensive Care Plan (CCP), see Supplementary File 2. The CCP was designed as part of the application for the organisation's accreditation, replacing the existing PCAP during the pilot period in these respective clinical areas. The research was part of the quality improvement initiative to guide the development of comprehensive care documentation that was responsive to patient and health professionals’ feedback.

### 3.3 Eligibility criteria

Participants were included in this study if they were:

- A nurse, doctor, or allied health professional.
- Over 18 years of age.
- Able to provide written and verbal informed consent.
- Patients who received care within the clinical divisions (irrespective of their health condition(s) or demographic characteristics).

### 3.4 Recruitment

A convenience sampling method (Etikan et al., 2016) was adopted to recruit all participants at each of the divisions. The participants in this project were not specifically targeted for a range of clinical and demographic diversity, because it was anticipated that there would be enough diversity within the sample given the broad range of clinical divisions involved in this project. Participants were assured that their comments would remain confidential and that all quotations would be deidentified to encourage free and open dialogue.

### 3.5 Data collection

All data collection was conducted in person by two experienced health service researchers (CP, KB), both were female, qualified registered nurses and senior researchers with experience of conducting qualitative research. Semi‐structured interviews were conducted in person or by telephone (mean time 30 min) and the focus groups were conducted in quiet private room (mean time 60 min).

The sample was determined in negotiation with the health service, who provided focus group meeting times for ward staff in cross over time periods. Each ward provided at least two patient interviewees and interviews depended on consent and availability. Given the open‐ended questions and different experiences, and diversity of staff and patients participating, broad understanding based on the planned sampling was expected and observed. The researchers continued sampling and analysing data until no new data appeared and all concepts were well‐developed, and no new data or codes were emerging, as agreed by all authors (CP, KB and CR).

A semi‐structured format was chosen to enable guided conversations around key issues informed by a topic guide (see Table 1) consistent with qualitative methods (Braun & Clarke, 2006). The discussions were fluid and flexible in nature, and all participants were encouraged to share beyond the established questions and probes. The qualitative data collection began with an opened ended, non‐directive question to encourage the participants to speak about their experiences in practice and care. Open‐ended probing questions were then used to elicit a greater detail of experiences shared by the participants.

**TABLE 1:** Interview topic guide questions

**Health Care Professionals** Can you tell me what you think about the Patient Care and Accountability Care Plan/Integrated Patient Risk Screening and Comprehensive Care Plan? Can you tell me about your current habit/practice of using the form in practice and completing it? How do you use the documentation in your daily duties for patients? Can you tell me how do you use the Patient Care and Accountability Care Plan/Integrated Patient Risk Screening and Comprehensive Care Plan to plan care? Intervene? And evaluate? patient care. What would help in developing shared care plans with patients in your place of work? How frequently do you consult the Patient Care and Accountability Care Plan/Integrated Patient Risk Screening and Comprehensive Care Plan to inform the ‘reality’ of what you did for your patients' care today, or last on shift? Is the nursing plan of care the same every day? or does it change? Can you tell me about your experience in practice to identify risks for preventable harms for patients in hospital? (pressure sore, falls risk, nutritional risk, VTE risk, cognitive impairment, self‐harm or suicide risk and aggression) Can you tell me, do some risks for preventable harm have more priorities than others? On reflection in practice, does some care get missed more often than others? What are these? What aspects of care are always delivered? What are your perceptions about the barriers/facilitators of using the Patient Care and Accountability Care Plan/Integrated Patient Risk Screening and Comprehensive Care Plan in routine practice? What is your overall perception of the Patient Care and Accountability Care Plan/Integrated Patient Risk Screening and Comprehensive Care Plan? How long does it take to complete the form? If you had to lose one thing on this form what would it be? If you had to add one thing on this form what would it be? What is the most important part of this form? Are there any aspects missing on this form? Do you think your nursing colleagues use this document to inform their nursing care? Do you think your allied health and medical team use this document? In what way? Are there times you priorities other tasks over this paperwork? What is it? And why?
**Patients** Can you tell me about your experience of care in hospital? Can you tell me what you think about the care that you have received from the health professionals involved in your care and treatment? Are there aspects of care that you would have liked that were not provided to you? What were these aspects? Were you involved and aware of being consulted in the decision‐making of your own individual needs for care, and how those needs would be met? Do you know if you care plan changes every day, or does it stay the same? Can you tell me what you think about the Patient Care and Accountability Care Plan/Integrated Patient Risk Screening and Comprehensive Care Plan? (show the patient the packs/care plan which patient signs) Have you seen this before? Can you please tell me what you think about this form to helping guide care for you? Have you been consulted in the shared completion of this care plan with your nurse? What is your overall perception of your Care Plan? What is the most important part of this form? Are there any aspects missing on this form? Are there any aspects that you don't like?

CP did not have any previous relationships with any of the participants; KB has been a nurse in the territory for 20 years and some participants may have had prior incidental contact such as working a shared shift. All interviews and focus groups were audio recorded using a digital recording device and transcribed verbatim by an external company. Reflective research notes were kept (by both CP and KB) on a computer file on the University's secure online database to capture initial impressions, thoughts and early interpretations of the data.

To ensure rigour, the following concepts were used: credibility, transferability, dependability and confirmability as identified by (Lincoln & Guba, 1985). The researchers conducting the qualitative data collection (CP and KB) ensured credibility by the audio recordings, noting thoughts and taking notes on reflective impressions immediately after each data collection. Findings were also presented back to the health service with opportunity for discussion. Transferability was addressed by providing a clear description of the setting and sample. Dependability in the project findings was addressed from the audit trail through the research notes used in the decision‐making process. Confirmability was ensured through clarification with open questions and repetitive questioning throughout the data collection, the reflective process after each data collection and peer discussion for data interpretation and verification. Trustworthiness is further supported using direct quotations, to show the connection between the data and results for the reader to interpret themselves. All quotations are provided verbatim with no identifiable information to protect confidentiality, and any editorial clarifications provided in [parenthesis]. To limit identification all nursing‐type participants are referred to as ‘nurse’ in quotations (Assistant in Nursing, Enrolled Nurse/Endorsed Enrolled Nurse, Registered Nurse, Manager and Educator).

### 3.6 Analysis

The qualitative analysis used an inductive thematic approach outlined in Table 2 (Braun & Clarke, 2006). Frequent discussions were held with the research team (CP, KB and CR) to ensure the established themes were accurately represented by the participant views.

**TABLE 2:** Phases of thematic analysis

Phase	Description
Familiarisation of data	Familiarisation of data was completed independently by CR, which involved reading and re‐reading the data. CR noted initial ideas and checked these with the post data collection reflective notes. CR, CP and KB also familiarised themselves with the data through the interview process, the completion of ‘post‐script’ field notes and further reading of the transcripts.
Generation of initial codes	CR, CP, and KB identified features of the data which were relevant to the overall aim of this project. Any discrepancies in codes were discussed openly in the research team to reach consensus.
Identifying themes	CR reviewed codes and began to organise data into preliminary themes according to similarities. At this stage, in response to project aims, all data were separated into categories of what was reported by participants to be useful (‘what works well/ ‘what they liked’) and what was not/ what they didn't like. All researchers discussed the preliminary themes to ensure a group consensus was reached.
Reviewing themes	CR, CP and KB further refined themes by ensure that the coded data extracts were accurately categorised into the appropriate theme. Coded extracts under each theme were re‐read to ensure it accurately represented the entire data set.
Defining and naming themes	CP developed a short description for each theme linked to the aim of this project.
Writing report	Relevant extracts linked to the overall aim of this project were identified, and a full report was written by CP. Contributions were received by CR and KB.

### 3.7 Ethics

This project received institutional approval from the Health Research Ethics and Governance Office (Project Number: 2020.QAI.00069). Written informed consent was received from all participants prior to the interviews and focus groups, and verbal consent was confirmed at the beginning of the audio recording. Participants could withdraw from the study at any time without stating a reason. Participants had the option to provide contact details to receive copies of the findings. We obtained verbal or written consent prior to all interviews. Data were anonymised for privacy and confidentiality reasons, and stored for a maximum of ten years.

## 4 RESULTS

A total of 16 patients or their family/carers from the different clinical areas consented to take part in a semi‐structured interview to share their experience of care (5 at Time 1 and 11 at Time 2), see Table 3. At baseline (time 1) data collection, there were 51 participants who consented to take part in the focus groups to share their experiences of using the Patient Care and Accountability Care Plan (PCAP), see Supplementary File 1. At time 2, there were 44 participants who consented to take part in the focus groups following the pilot of the Integrated Risk Screening and Comprehensive Care Plan developed as part of the accreditation processes, see Supplementary File 2. An overview of the clinical and demographic characteristics are detailed at Time 1 and Time 2 in Table 4. Notably, there was only one allied health professional who consented in this project, there were no other members of the multidisciplinary team (MDT) who consented to participate in this study across all the clinical sites. Ward characteristics of the pilot sites can be seen in Table 5. A total of 111 participants (12 patients, four family members/carers, 94 nurses and one allied health professional) consented to take part in this study. More than 30 h of audio recording was collected, approximately 700 pages of transcription.

**TABLE 3:** Overview of the characteristics of the patient participants at time 1 and time 2

Participants (Time 1) n5					
Division	Patient		Family / Carer		Total Time 1
	Gender		Gender		
	F	M	F	M	
Critical Care	0	1	0	0	1
Mental Health, Justice Health and Alcohol and Drug Services	1	0	0	0	1
Rehabilitation Aged and Community Services (acute)	0	0	0	1	1
Surgical	0	1	1	0	2
Total	1	2	1	1	4

Patients (Time 2) n11					
Division	Participant		Family / Carer		Total Time 2
	Gender		Gender		
	F	M	F	M	
Medicine	2	1	1	0	4
Mental Health, Justice Health and Alcohol and Drug Services	1	1	0	0	2
Rehabilitation Aged and Community Services (acute)	2	0	0	0	2
Rehabilitation Aged and Community Services	1	1	1	0	3
Total	6	3	2	0	11

**TABLE 4:** Overview of the characteristics of the healthcare professional participants at time 1 and time 2

Focus Group Results Summary (Time 1—PCAP)				
Focus Groups (n8)	Total participants	Demographic Variables		
Aged Care Rehabilitation Ward: *n* = 9 Emergency Department: *n* = 7 ENT and Plastic Surgery *n* = 7 Geriatric Unit: *n* = 8 Gastroenterology Ward: *n* = 5 Mental Health Short‐Stay Unit: *n* = 7 Orthopaedics, Oral Maxillo‐Facial Surgery: *n* =7 Radiation Oncology/Oncology *n* = 8	*n* = 51	Gender	Female	*n* = 47
			Male	*n* = 4
		Highest Qualification	School	*n* = 1
			Tafe / Hospital Trained	*n* = 4
			Bachelors	*n* = 21
			Honours	*n* = 1
			PG Certificate	*n* = 11
			PG Diploma	*n* = 7
			Masters	*n* = 4
			Masters by Research	*n* = 0
			Doctorate	*n* = 0
			Other	*n* = 0
			Missing	*n* = 2
		Position on Ward	Student	*n* = 1
			Admin	*n* = 1
			AIN	*n* = 0
			EN/EEN	*n* = 2
			RN1	*n* = 19
			RN2	*n* = 9
			RN3+ (educators/managers)	*n* = 16
			Allied Health	*n* = 1
			Missing	*n* = 2
		Length on Ward	<1 year	*n* = 9
			1–2 years	*n* = 16
			3–4 years	*n* = 5
			5–6 years	*n* = 12
			7–10 years	*n* = 3
			>10 years	*n* = 6
		Length in profession	<1 year	*n* = 2
			1–2 years	*n* = 7
			3–4 years	*n* = 7
			5–6 years	*n* = 5
			7–10 years	*n* = 8
			>10 years	*n* = 22

Focus Group Results Summary (Time 2—Post‐CCP Pilot)				
Focus Groups (n7)	Total participants	Demographic Variables		
Aged Care Rehabilitation Ward *n* = 5 Antenatal and Gynaecological: *n* = 7 Emergency Department: *n* = 11 Gastroenterology: *n* = 5 Geriatric Unit: *n* = 7 Mental Health Short‐Stay Unit: *n* = 4 Radiation Oncology/Oncology: *n* = 5	*n* = 44	Gender	Female	*n* = 39
			Male	*n* = 5
		Highest Qualification	School	*n* = 0
			Tafe / Hospital Trained	*n* = 5
			Bachelors	*n* = 13
			Honours	*n* = 0
			PG Certificate	*n* = 9
			PG Diploma	*n* = 9
			Masters	*n* = 3
			Masters by Research	*n* = 0
			Doctorate	*n* = 0
			Other	*n* = 0
			Missing	*n* = 5
		Position on Ward	Student	*n* = 0
			Admin	*n* = 0
			AIN	*n* = 1
			EN/EEN	*n* = 4
			RN1	*n* = 21
			RN2	*n* = 5
			RN3+ (educators/managers)	*n* = 8
			Allied Health	*n* = 0
			Missing	*n* = 5
		Length on Ward	<1 year	*n* = 8
			1–2 years	*n* = 9
			3–4 years	*n* = 12
			5–6 years	*n* = 1
			7–10 years	*n* = 1
			>10 years	*n* = 8
			Missing	*n* = 5
		Length in profession	<1 year	*n* = 3
			1–2 years	*n* = 7
			3–4 years	*n* = 6
			5–6 years	*n* = 0
			7–10 years	*n* = 5
			>10 years	*n* = 18
			Missing	*n* = 5

Abbreviations: AIN, assistant in nursing; EN/EEN, enrolled nurse/endorsed enrolled nurse; RN 1, Registered Nurse; RN 2, Senior Registered Nurse; RN 3, educator/manager.

**TABLE 5:** Pilot Ward demographics

	Geriatric Unit^iii^	Gastroenterology^ii^	Mental Health Short‐Stay Unit^vi^	Emergency Department^v^	Rehabilitation Aged Care^iii^	Radiation Oncology/ Oncology^iv^	Orthopaedics, Oral Maxillo‐Facial, ENT and Plastic Surgery^i^	Antenatal and Gynaecological^ii^ ^b^
1. General information								
Number of beds	26	24	6	86	26	26	28	15
Total Patients on 21 May 2020	20	24	6	218	23	24	24	10
Number of medical specialty teams	1	1	1	1	1	3	4	3
Bed to RN ratio (morning shift)	5.2	4.0	2.0	2.9	5.2	3.3	4.0	3.8
2. Risk profile per ward								
Proportion of patients with Falls Risk screening of >3	95%	29%	17%	N/A	96%	42%	29%	20%
Proportion of patients with Waterlow Score of >10	90%	50%	0%	N/A	91%	42%	38%	20%
Proportion of patients with malnutrition risk scores >2	25%	38%	0%	N/A	17%	42%	25%	N/A
Proportion of patients self caring with ADLs	0%	75%	67%	N/A	0%	54%	50%	80%
Latest audit completion percentage^a^	98%	98%	92%	N/A	100%	98%	96%	85%
3. Understanding complexity and acuity of the area								
Mean length of stay (days)	8	5	4	N/A	18	6	6	3
Mean number of AR‐DRGs for the ward in May 2020	31	41	20	N/A	17	51	60	43
Number of discharges for May (indication of turnover)	46	78	50	6206	28	77	82	200
Number of admissions from ED/emergency	93%	54%	100%	35%^c^	83%	71%	67%	20%

^a^ Combined completion rate of Falls Risk screening, Waterlow Assessment, Malnutrition Screening Tool and ADLs (excluding Malnutrition Screening Tool for ANW).

^b^ Adult patients included only (neonates excluded). RN, registered nurse; ADLs, Activities of Daily Living.

^c^ Emergency Department numbers provide the number of admissions that are admitted to a ward (the inverse of the rest of the row). Quotations are attributed to the overarching division: i. = Surgical, ii. = Medical, iii. = Rehabilitation Aged and Community, iv. = Cancer and Ambulatory Support, v. = Critical Care, and vi. = Mental Health, Justice Health and Alcohol and Drug services.

Based on the perspectives of patients' care experiences and healthcare professionals’ experiences of using healthcare organisational documentation in the context of managing preventable harms and delivering person‐centred care, the researchers identified three superordinate themes which were: (1) experiences of patient care, (2) what works well in practice, and (3) what does not work well in practice.

### 4.1 Theme ‘Experience of care’

The overarching theme of ‘experience of care’ consisted of the following subthemes: (1) ‘patients had high satisfaction with person‐centred care’; (2) ‘communication is important, but sometimes insufficient’; (3) ‘patients had variable involvement in their daily care plan’; and 4. ‘patients experience the lack of integration between multidisciplinary teams’.

#### 4.1.1 Patients had high satisfaction with person‐centred care

Most of the participants were very satisfied with the general nursing care and their hospital environment in the different clinical services and articulated very high praise of the nursing care experiences provided to them with resounding appreciation:

So, they remember me, and that can just help from an emotional, psychological point of view, just to feel people know who you are. Them coming back and say hello and smile. We have that level of familiarity. So that does help to make me feel more at home. (Patient, Medical Services, Time 2)

Participants expressed that the care provided to them from the nurses was tailored to meet their individual person‐centred needs and acknowledged that some nurse went that extra step to deliver exceptional care. To ensure that the patient's needs were being met, it was important that the nurses used active listening skills to understand what mattered most to the patients.

… they're doing a marvellous job to help cater for my needs, which is really good. (Patient, Mental Health, Justice Health and Alcohol and Drug Services, Time 1)

However, one participant articulated that to meet their own individual needs it was necessary to have a shared and unified holistic ‘bubble’ that included the nurse and the patient, but sometimes this was not always achieved:

The bubble is just I guess the separation point between the nurses and the patients. Usually there are nurses and doctors on staff, or maybe there’s a nurse on one particular shift who’s not on your wavelength, you usually get your message across. Yes. I’ve been here about five times, so I think over the time that I’ve been here I’ve learnt that. (Patient, Mental Health, Justice Health and Alcohol and Drug Services, Time 2)

#### 4.1.2 Communication is important and sometimes insufficient

While the participants were largely satisfied with their experience of care, communication issues were an aspect which caused concern and distress for some. Issues were related to communication during clinical triage and waiting for a bed, conveying the physical examination results with patients, and poor communication in delivering ‘bad news’ of new life‐limiting conditions.

I remember when someone was diagnosed in the ward, the doctor came in and said they found a mass, like pancreatic cancer. And they were saying she’d been diagnosed. She was like, okay, yes. Yes. And she got on the phone afterwards and she was like, I don't know what’s happening. (Patient, Surgical, Time 1)

Patients highlighted that this could be addressed by open‐ended opportunities for patients to ask questions or contribute to identifying their needs that were appropriate to their level of vulnerability:

To be able to just check in with them, like, okay, do you have any questions? And what about your husband, or wife, or your daughter, or son, would you like us to contact them, is often really helpful too. I've seen that so often where, especially someone elderly, or if English isn't their first language. It’s so important to have their family involved. And that can be the difference between everything for them. For the whole stay. Them knowing what’s happening and feeling more secure and all of that. (Patient, Surgical, Time 2)

Medical jargon was concerning to patients because they did not understand what their diagnosis meant, or what was going to happen in terms of their next steps in their care and treatment. Patients also highlighted that they needed to advocate for their own information needs, and this was a source of frustration.

#### 4.1.3 Patients had variable involvement in their daily care plan

There were mixed experiences of the patient's participation in their daily documented care plan across all the clinical sites. Most participants did not know what their daily care plan looked like, nor were they asked to sign this care plan.

No, I don't remember seeing that. (Patient, Rehabilitation Aged and Community Services, Time 2)

Most of the participants would not have wanted to sign the care plan daily either had they been asked to, if they were fully informed about their care and treatment.

I don’t know whether there’s really anything necessary that I need to see as long as I’m happy with the level of care that I receive. I know some people possibly benefit from looking through these and having this like a structure, I guess, for themselves, whereas I’m quite happy to go with the flow a little bit more. (Patient, Mental Health, Justice Health and Alcohol and Drug Services, Time 2)

Overall, participants felt involved and informed about their daily plan of care. Nurses provided them with the opportunity to ask questions, seek clarification and explanations when handover communication occurred at the patient's bedside. However, not all patients felt as informed of their care during nurse handovers when they did not happen at the patient's bedside.

You go and eavesdrop at handover so you can hear about what's happening with your own care. (Patient, Medical Services, Time 2)

#### 4.1.4 Patients experience the lack of integration between multidisciplinary teams

It became apparent that patients experienced issues with a lack of MDT integrated care across their journey. There were issues with patients having to tell their clinical history and story multiple times when they transitioned from one clinical area to another. This underscores the importance of having an integrated MDT document and in keeping with the nurses' experiences, devoid from duplication to ensure optimal care coordination.

What gets frustrating for every patient is you have to repeat the story over and over again to different people. So, you've done it multiple times down in ED because each person is starting from the front desk has to repeat the story. And then the nurse, and then the doctor, and then the specialist doctor. And then, sometimes another person they've referred. And then the nurse at the ward. And then they send the doctor in at night, or whatever, to see you. And you have to start all over again. (Patient, Medical Services, Time 2)

It was important that patients experienced clear communication in care and treatment across the MDT, which did not always happen well. Patients experienced a lack of time to understand and comprehend what the medical team were telling them, which was viewed as a very small window of opportunity for which they were often unprepared to ask questions and seek clarifications for their understanding.

I also think that people don't realise that, often, when they see the doctor in the morning, that little window, that might be the only time they see them. (Patient, Medical Services, Time 2)

Often the communication in care and treatment plans was completely absent in the MDT with the nurses relying on the patient for important updates on their clinical management.

Yes, actually, that happens a lot. Nurses will ask us what’s going to happen. So, have you seen the doctor, what did they say? Are you going to have this test done today? And I’ll say whatever. And they’ll say, I’ll go and check. It’ll be in the notes, and whatever. So, they know that they can check up. They're just as keen to hear. (Patient, Medical Services, Time 2)

#### 4.1.5 Theme ‘What works well in practice’

The overarching theme of ‘what works well in practice’ involved the following sub‐themes: (1) ‘structure of the documentation needs to be efficient’; (2) ‘ISBAR (introduction, situation, background, assessment, recommendation) valued for handover’; (3) ‘helpful prompting for clinical decision‐making is valued’; and (4) ‘direct patient care is always prioritised’.

#### 4.1.6 The structure of the documentation needs to be efficient

Nurses across the clinical areas shared several positive attributes of clinical documentation in practice. For the most part, nurses valued: documentation structure/layout that was clear and easy to follow, enabled the ability to retrospectively look back on the patient's clinical history in a stepwise approach and being able to trigger timely referrals based upon individual risk assessment for different domains of patient care.

… so we’ve got staff coming in and out, we’ve got staff who haven’t been exposed to that particular patient, so this (daily care plan) would be really good for them. Because you’ve been off for five days or whatever, and then you come back on, and patients have been discharged, and you’ve got a new patient. So, then you can look back … so that’s really good. (Nurse, Aged Care Rehabilitation Ward, Time 1)

The ‘shift priority box’ (a small open text section at the top of the daily care plan) on the structure of the documentation was viewed as slightly enhancing patient focused care, because the other existing documentation was largely based on a bio‐medical model for managing preventable harms and was inflexible and rigid. Nurses articulated that having the open space for the patient's shift priority aided patient handover, informed daily care plans, was related to clinical decision‐making, and nurses perceived that this helped them to ensure that nurses tasks were not left undone and unattended to between shift handovers.

Sometimes [the shift priority box] it’s helpful for handover. Like you use this [the shift priority box] when doing shifting because you can write the plan. It’s a good thing they’ve got some space to write the plan. (Nurse, Radiation Oncology/Oncology, Time 1)

Following the introduction of the new pilot documentation (CCP) (Supplementary File 2) at time 2, the nurses articulated that the new documentation assisted them to identify the needs of their patients, which was an improvement from the previous documentation used in practice. Some of the participants perceived that the new pilot documentation facilitated them to take a more holistic view to the development of shared care plans conducted with the patient.

I like that it has given me more insight into my patients’ care from a holistic point of view. Again, it’s taking me a lot longer to fill it out, but I then feel more confident in providing thorough care to my patient. (Nurse, Aged Care Rehabilitation Ward, Time 2)

However, consistently nurses expressed that the completion of revised documentation at Time 2 was more time intensive on their workloads compared to PCAP at Time 1, which added pressure to their already busy shifts.

Nurses highlighted that the consistent development of the nursing care plan through the trajectory of the patients' journey from admission and to different wards was important. That what why nurses valued the ISBAR format in documentation.

#### 4.1.7 ISBAR is valued for handover

Across the organisation nurses identified concerns about a lack of standardisation during the process of patient handovers within, and between, clinical areas. All nurses articulated the importance of implementing standardised patient handovers (both verbally and written) using the ISBAR format because of safety issues and risks with missed communication during clinical handovers of patients from different areas of practice.

I guess the other issue is that everywhere, everyone, handovers care differently. Some areas … don’t have any reference to the tool [ISBAR] … it becomes very tricky. (Nurse, Orthopaedics, Oral Maxillo‐Facial Surgery, Time 1)

Staff were supportive of documentation structures that continued the nursing plans of care from one area of the hospital to another, and forms that explicitly included ISBAR helped them to maintain patient safety.

#### 4.1.8 Helpful prompting for clinical decision‐making is valued

Nurses acknowledged that the previous PCAP documentation was a ‘tick and flick’ process to care documentation. However, nurses did find value in the daily structured assessments which helped them to trigger timely care referrals and interventions for those in their care, and the short time frame to complete. They did raise issues of accuracy of these ticks, in terms of consistency with the patient status. Irrespective of the clinical area of specialty all nurses experienced very busy shifts, heavy workloads, and therefore, they valued the daily prompt reminders for patient care, for example wound, stoma, and peripherally inserted central catheter (PICC) care. However, many nurses also identified that these are fundamental aspects of nursing care which they continued to deliver without the daily prompt reminders.

It’s all about what we should be doing, anyway, I guess. And it is a great prompt, because you do get busy, and you do forget, oh, have I gone in and have I looked at that wound, or have I rolled that patient? It’s been four hours since … It is a great prompt, but at the same time, I guess it then maybe stops us thinking clinically a little bit, because we’re relying on the form. (Nurse, Aged Care Rehabilitation, Time 1)

Nurses valued the overview of the patients' needs in relation to mobility, and other risk assessments but articulated that all patient assessments are driven by clinical judgement and professional nursing expertise:

So, in our clinical judgement, yes, this person may look like they’re a falls risk, but they’re actually not or they don’t have all the usual things that you’d expect, but they’re still a falls risk. Patients go around with no shoes on their feet or their shoes are hanging off them, things like that, they’re not over 65, they’re not Aboriginal and they’re not polypharmacy, because they’re not taking any medication, but they’re still a falls risk. (Nurse, Mental Health Short‐Stay Unit, Time 2)

Also ensuring that documentation had space to capture the nurse decision‐making was valued by nurses.

#### 4.1.9 Direct patient care is always prioritised

All nurses stated they prioritised direct patient care over the time required to complete the nursing documentation, even if this meant frequently staying on shift unpaid to complete what they articulated as ever‐increasing volumes of nursing documentation required. At both time points, nurses viewed the inability to complete their documentation, in a timely fashion, as a concern professionally because of the inherent legal requirements to evidence all aspects of care delivery in their practice.

Like, for me, as I do more hands‐on and still my documentation has to be second [priority], to be honest. (Nurse, ENT and Plastic Surgery, Time 1)

The tension of whether to document what was done, or just focus on doing what needs to be done, was problematic at all levels in the organisational documentation. For example, a manager identified that the documentation, including high scores on audit of that documentation, was needed to justify nursing work.

Well, basically, I [clinical nurse manager] know that you [as a ward team] do a great job with the patients, and then after one month, or two months later, we don’t have evidence of what you did last month. (Nurse, Aged Care Rehabilitation Ward, Time 1)

The pressure of documentation, or missed documentation, was something that kept nurses awake at night, with one even reporting returning to work after hours.

And it’s more about pressure on nurses about documentation … forgetting to record something, waking up in the night and thinking I haven’t recorded this, I have to go to ward and record it. (Nurse, Radiation Oncology/Oncology, Time 1)

#### 4.1.10 Theme ‘what does not work well in practice’

What does not work well in practice' encompassed the following subthemes: (1) ‘patient signature on the daily care plan is not valued’; (2) ‘multidisciplinary involvement is not facilitated by the current documentation’; (3) ‘excess duplication of paperwork’; and (4) ‘person‐centred goals are valued but not captured’.

#### 4.1.11 Patient signature is not valued

Across the healthcare organisation there is a requirement for all patients to sign their daily care plan. However, all nurses regarded this process to be unhelpful. Consistently, nurses reported that patients did not want to sign their daily care plan.

I find a lot of patients actually don't want to sign it, believe it or not. They say, I don't want my signature on this every day. (Nurse, Aged Care Rehabilitation Ward, Time 1)

Other considerations were required in this context for those patients who are involuntarily detained under the Mental Health Act or for those with cognitive impairment which made obtaining a daily patient signature inappropriate in practice. Nurses found this request required a delicate balance when trying to establish rapport with patients, particularly for the vulnerable, and was even potentially harmful.

… I would probably say like 75% of our consumers are involuntarily detained. If we were to go and write down their provisional diagnosis … experiencing psychosis or drug‐induced psychosis and we write it on there and ask them to sign it, we’re risking escalation. (Nurse, Mental Health Short‐Stay Unit, Time 1)

Nurses valued the development of rapport with their patients and avoided documentation if it was going to create a barrier to that rapport. At times, that rapport was the critical component of the health intervention being provided. Nurses continually partnered with their patients to involve them in their care throughout their shifts, including nursing handovers, without the organisational requirement of obtaining a signature to document involving patients in their care.

And at handover, we ask (the patient) is there anything else you want to add to the handover or anything like that. If they’ve got any questions. (Nurse, Radiation Oncology/Oncology, Time 2)

Nurses also articulated that their MDT colleagues are not required to ask their patients to sign their daily plan of care as a process of evidencing partnership or consent.

I haven't seen any doctor's plan where the patient is being signed. They are also making a plan about the patient treatment plan. (Nurse, Geriatric Unit, Time 1)

If obtaining the patient's signature was continued to be expected at the organisational level, then nurses wanted to have the ability to clearly document the reasons why the patient was unable to sign, or if the patient did not want to sign their care plan in keeping with the patient's own preferences for care. This reinforcement of the nursing decision‐making role as a partner in care needed signifiers within the documentation to facilitate their active and autonomous role in care.

#### 4.1.12 Multidisciplinary involvement is not facilitated by current documentation

All nurses viewed clinical documentation as a core component of nursing work, and thus they would like to see collaboration, review and input from the wider MDT (doctors and allied health professionals) in keeping with collaborative MDT models of care. However, the perception was that the document at both time points were not used by other disciplines across the organisation.

Nobody outside of nursing uses it at all. (Nurse, Radiation Oncology/Oncology, Time 1)

This was echoed by the lack of non‐nursing participants in this project. Other issues were triggered in the nurses' experiences of completing the documentation which had clear implications for a lack of MDT involvement, which resulted in a waste of nurses' valuable time and effort. The participants also highlighted that there were duplicate forms and items in the documents. Participants emphasised that the form may not have information other professionals considered relevant to their work. To the nurses, this disconnect reinforced the perception that the forms were completed for auditing and paperwork purposes only, rather than the creation of MDT person‐focused care.

… this form is the whole responsibility of the nurses. None of the other multi‐disciplinary team is involved in this form. (Nurse, Gastroenterology, Time 1)

#### 4.1.13 Excess duplication of paperwork

The sheer volume of paperwork in practice was problematic for all nurses. Most nurses relied on the progress notes to document the care they provided to their patients and to keep abreast of any physical and psycho‐social updates on individuals in their care.

You've got your four care plans.

You've got your notes.

Hourly rounding sheets.

And then, like syringe drive checks.

Bedside rounds

You've got your white notes. You've got these as well. (Nurse, Radiation Oncology/Oncology, Time 2)

The duplication was seen as time‐consuming and inefficient, and increased the level of cynicism felt towards required paperwork in general:

… a double job because I have to do progress notes, like detailed progress notes and I have to do detailed care plan and you are very busy with patients and everything. (Nurse, Geriatric Unit, Time 2)

This paperwork duplication was perceived to take nurses away from their primary task, which was to meet patient's needs.

That's why I call it ‘paper care’, not ‘patient care’. (Nurse, Geriatric Unit, Time 1)

#### 4.1.14 Person‐centred goals are valued but not captured in current documentation

Nurses were passionate about tailoring the care provided to their patients throughout all clinical areas. Both sets of documentation (at both time points) were not always conducive to aligning daily goals of care with patient's needs and preferences of care. Time constraints were an issue which nurses perceived as a barrier to meeting patient expectations and the individual needs of those in their care. The lack of space in the documentation also did not allow for the plans to progress or be captured in a sufficient manner. Nurses raised that in the organisation's attempt at being comprehensive in care, they were in fact not. This is not a new issue, that by trying to standardise care by its very nature was opposing ‘individual care’:

So, when we think about the care plan, the plan would be different [for each patient], or the goals are different, or interventions are different, so [the] care plan should be made according to the needs of the patients rather than generalising it. (Nurse, Geriatric Unit, Time 1)

A further consideration, which impacted person‐centred care, was the timeframes imposed by the organisations for the risk assessments to be completed, namely the 4‐hour and 8‐hour structures were problematic in practice. Most nurses reported issues from a clinical and professional standpoint in relation to the timeframes, especially related to when the patient risk assessments were completed, referrals, interventions delivered and follow‐up evaluation of person‐centred outcomes.

… the time frames are a big issue. Mainly because there’s no way to tell which tick boxes were done at which time without writing the date and time in every single box? And I’ve worked it out, there’s 55 questions. We can’t write the time and date 55 times. (Nurse, Geriatric Unit, Time 2)

Nurses were concerned about issues related to accountability for care and follow‐up of outstanding patient interventions and referrals being left undone without proper nursing and risk assessment. It was also important for staff working in ED to have their own section of the documentation because it was not feasible, or practical, for ED nurses to complete all the assessments during the patient's stay in emergency, given the fast‐paced and life‐threatening priorities that ED nurses continually face when providing care to their patients within this service.

… if you get a patient in the afternoon, they’re confused, they can’t give you the answers, their family is not coming till the next morning. You know we probably need 24 hours really to fill out a good quality proper care plan. (Nurse, Geriatric Unit, Time 2)

## 5 DISCUSSION

This study has identified that irrespective of the type of documentation being used in practice there were important short‐comings in relation to care coordination with a lack of MDT involvement in the development of person‐centred care and risk assessment practices, from both the patient and nursing perspectives. These challenges have also been reported elsewhere (Jweinat et al., 2013; Sharp et al., 2018). When healthcare information is collected multiple times on different records from MDT members, the integrity is compromised, contributing to inefficient use of limited resources and patient safety issues, and ultimately negatively impacts patient care experiences. One of the central benefits of good clinical care documentation is that it should facilitate more structured and focused communication between all MDT professionals. All clinical records are an essential tool for communication within, and between, clinical teams and they must reflect the patient's journey. Integrative MDT communication should importantly inform other healthcare professionals about the care/treatment which has been provided, and what care is being planned. Each individual patient record should accurately communicate within a healthcare team a ‘complete patient journey’. There is increasing awareness among healthcare providers that they must consider their services from the perspective of the patient. This study identified problems with care coordination, ineffective documentation of the ‘complete patient journey’ due to challenges of a lack of MDT integrated and shared record‐keeping processes. This issue was clearly articulated from the patient's perspective because they had to repeat their histories multiple times across their hospital journey, which was frustrating and resulted in a suboptimal care experience.

This study also highlighted ongoing issues around duplication of documentation, an issue widely acknowledged within the nursing profession (Cooper et al., 2021; Olivares Bøgeskov & Grimshaw‐Aagaard, 2019). Duplication of documentation or redundancy of items within the clinical document was reported to be problematic this study, irrespective of the type of document being used in practice. Fundamentally, excessive documentation is time‐consuming and takes nurses away from providing direct patient care, and the nurses referred to this as ‘paper care and not patient care’, which caused reduced satisfaction in the nursing process. The experiences of nurses in this study have been identified elsewhere and remains problematic in contemporary healthcare (Cooper et al., 2021). While the burden of clinical documentation is acknowledged, it is understudied with a lack of robust measurements in both inpatient and ambulatory settings to objectively quantify the issue and should be a future focus for further research (Moy et al., 2021).

The experience of person‐centred care was delivered well from both the nursing and patients accounts in this study. However, the documentation used in practice did not lend itself to capture person‐centred care plans. Specifically, this study identified both formal and informal aspects of partnership in the delivery of person‐centred care in addressing the needs of patients. The findings identified caring interactions between nurses and patients which were built on empathy, confidence and trusting relationships. Patients articulated that they felt informed of their plan of care and could ask questions and were listened too, particularly during nursing handovers. However, the organisational formal requirements to evidence partnership in delivery person‐centred care through daily patient signatures did not work well in practice. Patients’ themselves did not see value in signing a daily care plan, because they were continually informed of the nursing process and updated on their daily plan of care. Nurses also identified that obtaining a daily patient signature on the care plan document was not helpful and appeared to be tokenistic in nature. Nurses continually informed patients of their care, and not all patients were able to sign the care plan, that is patients affected by cognitive impairments. They both perceived that person‐centred care was achieved through informed discussion and agreement, and negotiation, and often included professional expertise and knowledge in the presence of a trusting and empathetic relationship.

As part of the cyclical organisational response to staff feedback in a quality improvement cycle, the health service further adapted the forms using the findings of this study and can be seen at Supplementary File 3.

### 5.1 Limitations

The patients were invited to take part in the study through first contact with nurses within each respective clinical site. Patients who agreed to participate in the interview might have been more attentive to positive care experiences and eager to talk about their experience. Therefore, this may have resulted in capturing more positive accounts of person‐centred care. However, to the best of our knowledge, this is the largest qualitative study to date in the important clinical area of documentation and multidimensional risk assessment and care plan strategies. We applied several approaches to ensure the trustworthiness in data collection and analysis which are a strength to this study. This study was conducted in one large healthcare organisation, which might impact on the transferability of the findings to other contexts.

## 6 CONCLUSION

Efficient and streamlined documentation systems should herald feedback from nurses to address their clinical workflow needs and can support, and capture, their decision‐making that enables partnership with patients to improve the individualisation of care provision. Nurses prioritised direct patient care over documentation requirements, but efficient documentation structures are valued by nurses when they can quickly inform their patient focused care.

## 7 RELEVANCE TO CLINICAL PRACTICE

Multidimensional risk assessment and care plan documentation strategies can reinforce person‐centred care and guide nurses in evidence informed decision‐making to reduce the risks of hospitalisation. However, the integration of effective MDT involvement in clinical documentation was problematic and resulted in unmet supportive care from the patient's perspective. Patients appeared to value the caring interactions and human connectedness more than the prescribed aspects of documenting agreed goals and care planning. Further research is needed to explore the barriers and facilitators of MDT involvement in healthcare documentation.

## CONFLICT OF INTEREST

None to declare.

## AUTHOR CONTRIBUTIONS

**Catherine Paterson**: Conceptualisation, methodology, validation, formal analysis, investigation, resources, data curation, visualisation, project administration, funding acquisition interpretation, writing original draft, writing—reviewing and editing, overall supervision. **Cara Roberts:** Formal analysis, interpretation, writing—reviewing and editing. **Kasia Bail:** Conceptualisation, methodology, validation, formal analysis, investigation, data curation, interpretation, writing original draft, writing—reviewing and editing.

## ETHICAL APPROVAL

Project Number: 2020.QAI.00069.

Consent to participate: Written informed consent obtained from all participants.

Consent for publication: Written informed consent obtained from all participants.

## Supporting information

Table S1

Supplementary Material

Supplementary Material

Supplementary Material

## Data Availability

The data that support the findings of this study are not publicly available due to ethical restrictions.
