# Supplementary material for: ‘Paper care not patient care’: Nurse and patient experiences of comprehensive risk assessment and care plan documentation in hospital
Source: J Clin Nurs. 2022 Mar 29;32(3-4):523–38. doi: 10.1111/jocn.16291 (PMC10084263; doi:10.1111/jocn.16291)
Supplement: Supplementary file 1 — Table S1 [file JOCN-32-523-s002.docx]

**Supplementary Table 1.** Consolidated Criteria for Reporting Qualitative Studies (COREQ) Checklist

| **No. Item** | **Description** | **Reported on page** |
| --- | --- | --- |
| **Personal Characteristics** |  |  |
| 1. Interview/facilitator | The author who conducted the interviews. | Page 7 |
| 1. Credentials | The researcher’s credentials. | Page 7 |
| 1. Occupation | The interview’s occupation at the time of the study. | Page 7 |
| 1. Gender | Male, female or non-binary. | Page 7 |
| 1. Experience and training | Experience and training of the researcher. | Page 7 |
| **Relationship with participants** |  |  |
| 1. Relationship established | Relationship prior to study commencement. | Page 7 |
| 1. Participant knowledge of the interviewer | Knowledge about researcher. | Page 7 |
| 1. Interviewer characteristics | Characteristics reported about the interviewer. | Page 7 |
| **Theoretical framework** |  |  |
| 1. Methodological orientation and theory | The methodological orientation underpinning the study. | Page 5 |
| **Participant selection** |  |  |
| 1. Sampling | Method of participant selection. | Page 5 |
| 1. Method of approach | How participants were approached. | Page 6 |
| 1. Sample size | Number of participants in the study. | Page 8 |
| 1. Non-participation | Number of participants who refused to participate or dropped out. | Page 8 |
| **Setting** |  |  |
| 1. Setting of data collection | Location of data collection. | Page 5, Table 5 |
| 1. Presence of non-participants | Presence of other individuals at the time of data collection. | Page 5 |
| 1. Description of sample | Important characteristics of the sample. | Page 6, Table 3 and 4 |
| **Data collection** |  |  |
| 1. Interview guide | Interview guide and prompts used. | Page 7 and 8, Table 1 |
| 1. Repeat interviews | Statement of whether repeat interviews were conducted. | Page 6 |
| 1. Audio/visual recording | Type of interview recording. | Page 7 |
| 1. Field notes | Description of field notes made during or after the interview. | Page 7 |
| 1. Duration | Duration of the interviews. | Page 7 |
| 1. Data saturation | Discussion around data saturation. | Page 7 |
| 1. Transcripts returned | Return of transcripts to participants. | Page 8 |
| **Data analysis** |  |  |
| 1. Number of data coders | The number of data coders who coded the data. | Page 8, Table 2 |
| 1. Description of the coding tree | Description of coding tree. | Page 9, Figure 1 |
| 1. Derivation of themes | Identified in advance or derived from the data. | Page 9, Figure 1 |
| 1. Software | Software used to manage the data. | Page 7 |
| 1. Participant checking | Feedback from participants. | Page 8 |
| **Reporting** |  |  |
| 1. Quotations presented | Participant quotations presented to illustrate the themes. | Page 9 -22 |
| 1. Data and findings consistent | Consistency between data presented and the findings. | Page 19 -22 |
| 1. Clarity of major themes | Major themes clearly presented. | Page 9-22, Figure 1 |
| 1. Clarity of minor themes | Description of minor themes or categories. | Page 9-22, Figure 1 |
