# Supplementary material for: ‘Paper care not patient care’: Nurse and patient experiences of comprehensive risk assessment and care plan documentation in hospital
Source: J Clin Nurs. 2022 Mar 29;32(3-4):523–38. doi: 10.1111/jocn.16291 (PMC10084263; doi:10.1111/jocn.16291)
Supplement: Supplementary file 2 — Supplementary Material [file JOCN-32-523-s004.pdf]

PATIENT CARE AND ACCOUNTABILITY PLAN

Complete details or affix label

URN: ____________

Family name: ____________

Given names: ____________

DOB: ____________ Sex: ____________

SECTION ONE - Discharge Planning

Estimated Date of Discharge (EDD)

Date: ______ / ______ / ______ Day of week for D/C: ____________ Time: ______

Patient informed of EDD? ☐ Yes ☐ No Date: ______ Time: ______

Patient informed of any change? ☐ Yes ☐ No Date: ______ Time: ______

Reason for EDD change: ____________

Notified of possible use of D/C lounge: ☐ Yes ☐ N/A Date: ______ Time: ______

Is complex discharge planning required? ☐ Yes ☐ No

If **yes, WHY?** ____________

Note that this section is not the formal referral to the service required. Use usual referral processes

	Referred	In progress	Ready for D/C		Referred	In progress	Ready for D/C
**PT**	☐	☐	☐	**SW**	☐	☐	☐
Date/Time				Date/Time			
**OT**	☐	☐	☐	**Other:**	☐	☐	☐
Date/Time				Date/Time			
**DLN:**	☐	☐	☐	**Other:**	☐	☐	☐
Date/Time				Date/Time			

Discharge destination: ☐ Home ☐ Other (specify): ____________

Transport ☐ Self ☐ Other (specify): ____________

☐ Transport booked Date: ____ / ____ / ____ Time: ______

Checklist

DC summary completed ☐ Yes

Patient to wait for copy of D/C summary? ☐ Yes ☐ No, will mail out

Medical certificate provided? ☐ Yes ☐ N/A

D/C information/education sheets provided ☐ Yes ☐ N/A

Nursing transfer documentation complete (if applicable) ☐ Yes ☐ N/A

Pathology Pre DC bloods required? ☐ Yes ☐ No Details: ____________

Pharmacy ☐ Script complete ☐ Script sent to Pharmacy ☐ Medications ready

☐ Own medications returned

Discharge ☐ All films/documents/belongings etc, returned ☐ IV access removed (if applicable)

☐ Transferred to DC Lounge Time of transfer: ____________

Signature of discharging staff member	Print name	Designation	Date/Time of D/C

PATIENT CARE AND ACCOUNTABILITY PLAN

65212

SECTION TWO – Admission Overview   If able, ask the patient and or carer to fill in blue areas.

Provisional Diagnosis: ______________________________

____

Admit Date: ____ / ____ / ____   Admit time (24hrs): ____________   Ward: ____________

Use if patient transferred to other ward: Date T/F: ____/____/____   Receiving Ward: ____

Date T/F: ____/____/____   Receiving Ward: ____   Date T/F: ____/____/____   Receiving Ward: ____

ID band in place & correct ☐ Yes   Allergy band in place and noted on medication chart ☐ Yes ☐ N/A

Other Alerts (specify): ______________________________

NOK details correct on admission form ☐ Yes   **if not correct, update**

Important contact/s (other than NOK): ______________________________

Identify as Aboriginal or Torres Strait Islander? ☐ Yes ☐ No **ensure noted in patient details**

Preferred Language: ____________   Interpreter ☐ Yes ☐ No   Date/time notified: ____/____/____

Directives and Legal: Does the patient have a medical advance care direction documented in their notes, e.g. NFR order? ☐ Yes ☐ No   **If yes, ensure it is clearly identified and easy to locate**

Tick if any legal document/directives listed below are in place and provide details, e.g. key contacts:

☐ Advance Care Plan/Statement of Choices   ☐ Health Direction (including blood transfusions)

☐ Enduring Power of Attorney   ☐ Mental Health Act Treatment Order   ☐ Guardianship Orders

☐ Other (e.g. AVO, domestic violence order): ______________________________

Details: ______________________________

Ensure a copy of any directive or legal document is on file in the patients notes

For more information, contact the Respecting Patient Choices Team.

Supportive Aids: Dentures: ☐ Yes ☐ No Specify type: ______________________________

Visual Aids ☐ Yes ☐ No Specify: ____________   Hearing Aids: ☐ Yes ☐ No ☐ Left ☐ Right

Mobility Aids ☐ Yes ☐ No Specify: ____________   **Ensure w/chair and cushion is within pts reach**

Specialised Equipment e.g. CPAP ☐ Yes ☐ No Specify: ______________________________

Preadmission living status: Do you: ☐ Live alone ☐ Care for someone else

☐ Use home/community services   Other: ______________________________

Ward Orientation: Bathroom/toilet facilities ☐ Yes   Staff roles and uniforms ☐ Yes

Visiting hours ☐ Yes   No smoking policy ☐ Yes   CARE for Patient Safety program ☐ Yes

Use of mobile phone/computer/telephone/radio/TV/nurse call bell ☐ Yes

Patient's Rights and Responsibilities (pamphlet) ☐ Yes

If unable to orientate patient to above, state why: ______________________________

Valuables: ☐ With patient ☐ Sent home ☐ Secured in hospital safe ☐ Patient informed of valuables policy

Medications: ☐ Locked up ☐ Sent home   Patient supportive aids: ☐ With patient ☐ Sent home

Comment: ______________________________

Infection Prevention and Control Unit Alerts

1. Has the patient had a known MRO? ☐ Yes ☐ No   If yes, specify type: ____________

Screen patient according to hospital protocol and implement appropriate precautions

2. Is this admission for diarrhoea, flu or a surgical site infection? ☐ Yes ☐ No **If yes to any, notify IPCU**
3. Has the patient had Chicken Pox or been vaccinated for same? ☐ Yes ☐ No **If no/unsure and the patient is pregnant, notify IPCU**

Date swab taken (if required): ____/____/____   Date/time IPCU notified: ____/____/____ ____

Signature	Print name	Designation	Date/time

SECTION THREE - Assessments (continued)

3. Nutrition (Malnutrition Screening Tool – MST)

Use additional Malnutrition Screening Tool Form if additional rescreening required

	Circle Score	Date	Circle Score	Rescreen Date	Circle Score	Rescreen Date	Circle Score	Rescreen Date
1. Have you/the patient lost weight recently without trying								
No	0		0		0		0	
Unsure	2		2		2		2	
Yes *(how many kg?)*								
1-5kg	1		1		1		1	
6-10kg	2		2		2		2	
11-15kg	3		3		3		3	
>15kg	4		4		4		4	
Unsure	2		2		2		2	
2. Have you/the patient been eating poorly because of a decreased appetite								
No	0		0		0		0	
Yes	1		1		1		1	
**Total score**								
Patient weight (kg)								
Referral to Nutrition Department	☐ Yes ☐ No		☐ Yes ☐ No		☐ Yes ☐ No		☐ Yes ☐ No	
Nourishing Diet Commenced	☐ Yes ☐ No		☐ Yes ☐ No		☐ Yes ☐ No		☐ Yes ☐ No	
Food Chart Commenced	☐ Yes ☐ No		☐ Yes ☐ No		☐ Yes ☐ No		☐ Yes ☐ No	

Malnutrition Risk Score

MST Score = 0-1	MST Score = 2	MST Score 3-5 or two MST scores of ≥ 2
1. Continue current diet<br>2. Rescreen weekly	1. Call Nutrition Department and request nourishing diet<br>2. Re-screen weekly<br>3. Consider starting food chart	1. Call Nutrition Department and request nourishing diet and dietitian assessment<br>2. Commence food chart if patient unable to communicate oral intake accurately

Signature ____________ Print name ____________ Designation ____________ Date/Time completed ____________

4. Pressure Injury Risk Assessment (Waterlow[2])

☐ **Pressure Injury Information Provided**

Circle applicable score. Add total score. Several scores may be selected in some categories

Sex and Age		Skin Type and Visual Areas		Continence		Tissue Malnutrition	
Male	1	Healthy	0	Complete/Catheterised	0	e.g.	
Female	2	Tissue paper	1	Occasionally incontinent	1	Smoking	1
14-49	1	Dry	1	Catheter/incontinent of faeces	2	Anaemia	2
50-64	2	Oedematous	1	Doubly incontinent	3	Peripheral Vascular disease	5
65-74	3	Clammy	1			Cardiac Failure	5
75-80	4	Discoloured	2			Terminal Cachexia	8
80+	5	Broken	3				

Mobility		Neurological Deficit		Appetite		Build/Weight for Height	
Fully	0	(e.g. Diabetes, MS, CVA, Motor/sensory paraplegia)		Average	0	Average	0
Restless/fidgety	1			Poor	1	Above average	1
Apathetic	2			NG tube/fluids only	2	Obese	2
Restricted	3	Moderate	4	NBM/anorexia	3	Below average	3
Inert/traction	4	Moderate-severe	5	**Major Surgery/Trauma**		**Medication**	
Chair-bound	5	Severe	6	Orthopaedic – below waist, spinal	5	Cytotoxics	
				On table > 2 hrs (within last 48 hrs)	5	High Dose Steroids	
						Anti-inflammatory	4

10+ At Risk	15+ High Risk	20+ Very High Risk	Risk Score	
Implement prevention strategies within 2 hours	Implement prevention strategies within 2 hours	Implement prevention strategies within 30 mins		

If AT RISK for pressure injury, refer to Care Plan Section for intervention and management

Signature ____________ Print name ____________ Designation ____________ Date/Time completed ____________

SECTION FOUR - Patient Care Plan Complete appropriate Care Plan section for each shift

Date: ________ **Number of Days admitted:** ______ **EDD:** ________ **Ward:** ______ **Refer to page 1 for D/C planning** **Please ensure patient label is affixed to one side of each care plan**

**Handover Notes**<br>*Use this section to note points to be noted in handover, e.g. expected tests, guidelines*<br>*Use ISBAR to handover*	**AM**	**PM**	**ND**

	**Complete on Morning Shift or Shift of Admission**	**Variance** ☐ PM ☐ ND	**Variance** ☐ PM ☐ ND
**Clinical Incident Reporting**	Incident type: ________<br>________ ☐ **Riskman completed** ☐ **Entered in notes**	☐ **Riskman completed**	☐ **Riskman completed**
**Observations and Frequency**	**Vital signs:** Frequency: ______ **O_2:** Requirements: ______ ☐ **BGL:** Frequency: ______<br>**Weight:** Frequency: ______ **Date Due:** ______ ☐ **Weight noted on chart**<br>☐ **Other observations** (specify): ______	(note changes)	(note changes)
**Input** **Nutrition:**<br>*How long has your patient been fasting?*<br>**Intravenous:**<br>*Does your patient need IV access? Can it be removed?*	☐ Oral Specify diet, including restrictions: ______<br>Food assistance: ☐ Nil ☐ Full feed ☐ Set up ☐ Food chart<br>☐ NBM NBM reason: ______ No. days NBM: ______ ☐ TPN<br>☐ Enteral *(circle route)* NG / PEG / Other: ______ Feed type: ______<br>Line type/site: ____ Insertion date: ____ Dressing/resite due: ____ Cap due: ____<br>Line type/site: ____ Insertion date: ____ Dressing/resite due: ____ Cap due: ____<br>Line type/site: ____ Insertion date: ____ Dressing/resite due: ____ Cap due: ____	(note new lines and location)	(note new lines and location)
**Output**<br>**Fluid Balance Chart Required?**<br>☐ **Yes** ☐ **No**	**Urine:** ☐ Self Caring ☐ IDC/SPC Date of insertion: ______ ☐ Stoma<br>☐ Assist/Pan/Urinal ☐ Incontinent Abdomen measurement for continence aid size (cm): ______<br>**Drains:** Specify site/s and special orders: ______<br>**NG:** ☐ Free drainage with ______ hourly aspiration Special orders: ______<br>**Bowels:** ☐ Self Caring ☐ Assist/Pan ☐ Incontinent ☐ Stoma ☐ Stool Chart	**Fluid Balance Chart** ☐ Yes ☐ No	**Fluid Balance Chart** ☐ Yes ☐ No
**Venous Thromboembolism**	☐ Reassessed Patient at risk of VTE? ☐ Yes ☐ No ☐ Requirements noted on medication chart<br>**Notes** (e.g. compression stockings) ______	(note changes)	(note changes)
**Falls**<br>**Falls Risk Score** (assess daily and if condition changes and on D/C):<br>______<br>☐ Tick if falls education provided	**Do the following for ALL patients 'at high risk' of falls:** ☐ 'Falls risk' sign in place above bed<br>*Use Bed rails assessment matrix.* Rails ☐ UP ☐ DOWN ☐ Call bell within reach<br>**Choose at least 3 other interventions for pts at risk (ensure A/H referrals completed):**<br>☐ Hi-low bed ☐ Supervise in bathroom ☐ Walking aid within reach<br>☐ Adhere to toileting regime ☐ Bed/chair alarm ☐ Intentional/hourly rounding<br>☐ **ALERT** - tick if the patient on anticoagulant/s Falls focused medication review undertaken ☐ Yes ☐ No ☐ N/A	(note changes and reassess if required)	(note changes and reassess if required)
**Pressure Injury**<br>**PI present on admission?**<br>☐ **Yes** ☐ **No**<br>**Waterlow Risk Score** (assess daily & if condition changes): ______<br>☐ Tick if PI education provided	**Assess:** Skin Intact ☐ Yes ☐ No Pressure Injury site/s: ______<br>☐ Stage 1 ☐ Stage 2 ☐ Stage 3 ☐ Stage 4 ☐ Unstagable ☐ Suspected Deep Tissue Injury<br>**Interventions:** ☐ 2hourly turns ☐ 4 hourly turns ☐ Self Caring<br>Heels offloaded / suspension device used ☐ Yes ☐ No ☐ Active air cushion ☐ Active air mattress<br>Preventative foam sacral/heel dressing ☐ Yes ☐ No Specify where: ______<br>☐ Moisturise skin daily ☐ Nutrition Review<br>☐ Refer to Tissue Viability Unit ***Use Wound Care section below for any dressings***	(note changes and reassess if required)	(note changes and reassess if required)
**Wound Care**	**No. of wounds:** ____ **Locations/s:** ______<br>☐ Referred to tissue viability unit Date: ______ ☐ Wound assessment and management form	(note changes)	(note changes)
**Mobility/Manual Handling**<br>*Mobility changes? Use pg 3 & 5.*	**Lifting aid required:** ______ **Mobility aid required:** ______<br>**Staff Assist:** ☐ 1 nurse ☐ 2 nurses ☐ Self Caring ☐ Confined to bed	(note changes)	(note changes)
**ADLs**	**Hygiene:** ☐ Self Caring ☐ Shower ☐ Assistance required: ______<br>Other/notes /special cleanser required: ______<br>______ **Mouth Care:** ☐ Self Caring ☐ Assist	☐ Bedside equipment check complete	☐ Bedside equipment check complete
**Shift completing care plan** ☐ **AM** ☐ **PM** ☐ **ND** ☐ Bedside equipment check complete<br>______ ______ ______ ______ ______<br>Signature Print name Designation Date Time:<br>**Patient signature (if able):** ______ ☐ Tick if unable to sign		**Signature:** ______<br>**Print name:** ______<br>**Designation:** ______<br>**Date:** ______ **Time:** ______	**Signature:** ______<br>**Print name:** ______<br>**Designation:** ______<br>**Date:** ______ **Time:** ______

Complete details or affix label

URN: ______________________

Family name: ______________________

Given names: ______________________

DOB: ______________ Sex: ______________

PATIENT CARE AND ACCOUNTABILITY PLAN

SECTION THREE - Assessments (continued)

5. Venous Thromboembolism Risk Assessment

Use the Adult VTE Risk Assessment and Prophylaxis Guide to complete the VTE assessment and ensure the medication chart is completed

Risk of VTE present? ☐ Yes ☐ No

Patient already on anticoagulants? ☐ Yes ☐ No e.g. warfarin, enoxaparin, heparin, apixaban, rivaroxaban, dabigatran

Note: If yes to above and the pt is at risk of falls, ensure appropriate falls prevention interventions are commenced in the care plan

Pharmacological Prophylaxis and Mechanical Prophylaxis must be ordered and written on medication chart. Ensure this is documented in the daily care plan

Comments: ______________________

Signature	Print name	Designation	Date/Time completed

6. Mobility Assistance (on admission)

Use the following flow chart to assess mobility/manual handling requirements. When patient condition changes, ensure care plan is updated appropriately, e.g. after surgery.

☐ Tick if patient is bariatric/obese (>125kg)

ON BED
- Can pt comprehend and cooperate? → Yes / No
- Can pt maneuver their own body? → Yes / No
- No → Use a minimum of two staff members at all times → Slide sheets must be used

OFF BED
- Can pt comprehend and cooperate? → Yes / No
- Can pt maintain their own balance? → Yes / No
- No → Use a minimum of two staff members at all times → Lifting device must be used

Slide sheets required? ☐ Yes ☐ No Lifting device required? ☐ Yes ☐ No Prescribed bed rest ☐ Yes

If patient has a wheelchair and cushion, ensure they are within reach

Signature	Print name	Designation	Date/Time completed

7. Is there any other information about your health, health conditions, special circumstances or needs that you would like noted that will assist with your care? e.g. chemotherapy last 7 days, no blood products, pre-existing access lines, special care needs, recent overseas trip

☐ Yes ☐ No, if yes, specify:

Signature	Print name	Designation	Date/Time completed

Patient signature *(if able to sign)*: ______________________ ☐ Tick if unable to sign

PATIENT CARE AND ACCOUNTABILITY PLAN

Complete details or affix label

URN: ______________________

Family name: ______________________

Given names: ______________________

DOB: ______________ Sex: ______________

SECTION THREE - Assessments — Perform all assessments/screens on admission

1. Systems Assessment (on admission)

**Cognition**	*Ask the following questions.* 1. What is your age? 2. What is your date of birth? 3. What is the name of this place? 4. What is the current year?<br>*Did the pt answer ALL of the questions correctly?* ☐ Yes ☐ No<br>*If no, consider further cognitive/delirium assessment*
**Neurological**	☐ Alert and orientated ☐ Drowsy and orientated ☐ Confused ☐ Nil response<br>**Commence neurological chart if appropriate**
**Vital signs**	☐ Admission vital signs documented using MEWS scoring criteria
**Breathing**	☐ Breathing without effort ☐ Breathing with some effort ☐ Breathing with significant effort<br>Chest Ausculated ☐ Yes ☐ No If yes, describe: ______<br>Does the patient smoke? ☐ Yes ☐ No If a smoker, NRT offered ☐ Yes
**Circulation**	**Peripheries:** ☐ Warm and well perfused ☐ Cool ☐ Cold<br>**Central:** ☐ Warm and dry ☐ Cool ☐ Clammy ☐ Febrile
**Skin Integrity**	**Check skin:** ☐ Intact ☐ Broken - **Complete wound assessment and management plan**
**Oral Hygiene**	☐ Self Caring ☐ Assist ☐ Own teeth
**Urinary**	☐ Continent ☐ Incontinent ☐ Aids (e.g. pads): ______<br>UA completed ☐ Yes ☐ No ☐ IDC/SPC **Record insertion/change date on care plan**
**Gastro intestinal**	☐ No issues ☐ Reflux ☐ Nausea ☐ Vomiting<br>Bowels: ☐ Regular ☐ Irregular ☐ Continent ☐ Incontinent ☐ Stoma
**ADLs**	Are there any factors present that will affect ADLs? ☐ Mobility ☐ Vision ☐ Speech<br>☐ Respiratory ☐ Hearing ☐ Cognition ☐ Wounds ☐ Pain ☐ Swallow ☐ Other
**Sleep Pattern**	☐ No issues ☐ Issues, describe: ______
**Diet/Nutrition/ Alcohol/Drugs**	☐ Normal Diet ☐ Diabetic ☐ Diet requirement, describe ______<br>☐ Texture modified ☐ Fluids **Ensure nutritional risk assessment completed on page 4**<br>Does the patient consume alcohol/drugs? ☐ Yes ☐ No If yes, provide details: ______

Signature ______ Print name ______ Designation ______ Date/Time completed ______

2. Falls Risk Screening Assessment[1] — ☐ Falls Risk Information Provided

**Modified Stratify[1] Falls Tool** Document admission assessments here. Document reassessments in Care Plan			Score
1.	Fall: current admission	☐ Patient had fall/s during current admission or admitted as a result of a fall	3
2.	Fall: within 12 months	☐ Patient had fall/s in the last 12 months (from history)	1
3.	Cognition	☐ Patient is either confused, agitated, lacks insight or is impulsive	1
4.	Mobility	☐ Patient requires supervision or assistance with mobilising	1
5.	Impaired Balance	☐ Patient has impaired balance and/or hemiplegia	1
6.	Age	☐ Patient is 80 years old or older	1
7.	Toileting	☐ Patient is needing frequent toileting	1
8.	Vision	☐ Patient is visually impaired to the extent that everyday function is affected	1
9.	Drug/Alcohol	☐ Patient presented with drug/alcohol related problems	1
**A score of 3 or more is considered 'HIGH RISK'. Use the Care Plan to choose appropriate interventions and management**		**Risk Score**	

Signature ______ Print name ______ Designation ______ Date/Time completed ______
