# Supplementary material for: ‘Paper care not patient care’: Nurse and patient experiences of comprehensive risk assessment and care plan documentation in hospital
Source: J Clin Nurs. 2022 Mar 29;32(3-4):523–38. doi: 10.1111/jocn.16291 (PMC10084263; doi:10.1111/jocn.16291)
Supplement: Supplementary file 3 — Supplementary Material [file JOCN-32-523-s003.pdf]

Swallowing	< 8 hours No	Yes	ACTIONS	Initial
Do you have trouble swallowing your food, drinks or tablets?	☐	☐	Refer to Speech Pathologist and<br>Notify Medical Officer	

Alcohol, Tobacco and other Drugs	< 8 hours No	Yes	ACTIONS	Initial
Is the patient a regular smoker or has smoked in the past 30 days?	☐	☐	Offer NRT	
Does the patient drink > 6 standard drinks/session?	☐	☐	Initiate Alcohol Withdrawal Scale	
Does the patient use illicit or non-prescribed drugs in previous month?	☐	☐	Contact Drug & Alcohol Liaison Service.	
Is the patient on opiate replacement therapy?	☐	☐		

End of Life	< 8 hours No	Yes	ACTIONS	Initial
Is the patient:<br>☐ 65 years or older or<br>☐ 45 years or older if Aboriginal & Torres Strait Islander	☐	☐	If yes to both, refer to Medical Officer to conduct End of Life Screening Tool	
AND Does the patient present with 2 or more of the following:<br>☐ Poor or deteriorating health<br>☐ Previous unplanned hospital admission<br>☐ Life limiting illness or disability<br>☐ Family express concern about quality of life	☐	☐		
Would you be surprised if this person died in the next 30 days?	☐	☐	If **No**, refer to Medical Officer to conduct End of Life Screening Tool	

Social, Wellbeing, Disability	< 8 hours No	Yes	ACTIONS	Initial
Does the patient have any specific cultural or religious needs while in hospital?	☐	☐	Document details of specific needs	
Does the patient identify as having a disability requiring assistance in hospital?	☐	☐	Incorporate in patient's care plan.	

Existing services: ☐ No existing services ☐ Community nursing ☐ Home Help ☐ Meals on Wheels ☐ NDIS ☐ Other/s: ____________

Accommodation on Admission: ☐ Own Home ☐ Hostel ☐ Nursing Home ☐ Other: ____________

Access: ☐ Flat ☐ Stairs, how many: ______ ☐ Ramp ☐ Lift

Living arrangements: ☐ Lives alone ☐ Lives with family ☐ Lives with others ☐ Homeless

Carer Details: ☐ Patient is a carer ☐ Carer lives with patient ☐ Carer not living with patient ☐ No carer ☐ Other: ____________

How does the patient expect to travel home from hospital once well enough?

____

____

Ask the patient if there is any other information that is important for staff to know about themselves or family members to assist us providing better care.

☐ Unable to ask ☐ No suggestions

☐ Yes Details: ____________

____

Signature ______ Print name ______ Designation ______ Date/Time ______

DO NOT WRITE IN THIS BINDING MARGIN

35016(0320)

Integrated Patient Risk Screening - Adult

Identifying patients who are at risk of harm whilst in hospital and mitigating the risk for those patients is a core part of comprehensive care planning and treatment.

The Integrated Risk Screening Tool - Adult is to be used for all adult patients, excluding Maternity, admitted to Health Services. Risk screening should commence at the entry point of admission irrespective of location - Emergency Department, Direct Admission to Ward or Other (e.g. DOSA, Outpatient Clinic, Rapid Assessment Unit).

In the Emergency Department, risk screening is required for all patients admitted or requiring admission and for those patients identified as higher risk including:

- Age of 65 years and over; or 45 years and older for Aboriginal and Torres Strait Islander peoples
- Complex care needs
- With clinical conditions, co-morbidities and social circumstances suggesting a level of risk of harm.

THIS PAGE HAS BEEN LEFT BLANK
Do not write on this page

DO NOT WRITE IN THIS BINDING MARGIN

Complete details or affix label

URN: ____

Family name: ____

Given names: ____

DOB: ____ Sex: ____

INTEGRATED PATIENT RISK SCREENING - ADULT

Elimination	< 4 hours No	< 4 hours Yes	ACTIONS	Initial
Is the patient continent?	☐	☐	Record admission Urinalysis	
If no,<br>☐ Urinary incontinence ☐ Faecal incontinence<br>☐ Continence / Toileting aids required:<br>☐ Stoma ☐ Urinary Catheter			Document toileting aids required	
	< 8 Hours			
Last bowel movement? ____<br>Regularity of bowel movements: ____				
☐ Constipation ☐ Diarrhoea	☐	☐	Notify concerns to Medical Officer	

VTE	< 8 hours No	< 8 hours Yes	ACTIONS	Initial
Has a VTE risk assessment been completed & documented by the MO?	☐	☐	If No, refer to MO for VTE Risk Assessment within 24 hours	
If yes, has VTE Prophylaxis been prescribed commensurate with VTE risk identified	☐	☐	If No, refer to MO	

Nutrition and Weight	< 8 hours No	< 8 hours Yes	ACTIONS	Initial
Weight recorded on admission: ____ kg<br>☐ Unable to weigh → Estimated weight: ____ kg				
Is the patient > 120 kg	☐	☐	Source appropriate equipment	

Nutrition (Malnutrition Screening Tool – MST)

Use additional Malnutrition Screening Tool Form if additional re-screening required

Date:

1. Have you/the patient lost weight recently without trying?	
**No**	☐ 0
**Unsure**	☐ 2
**Yes** *(how many kg?)*	
1-5kg	☐ 1
6-10kg	☐ 2
11-15kg	☐ 3
>15kg	☐ 4
Unsure	☐ 2
2. Have you/the patient been eating poorly because of a decreased appetite?	
**No**	☐ 0
**Yes**	☐ 1
**Total score**	

MST score	Action
0 - 1	**Continue current diet**<br>Re-screen weekly
2	Call Nutrition Department and request nourishing diet<br>Re-screen weekly<br>Consider starting food chart
3 - 5	Call Nutrition Department and request nourishing diet and dietitian assessment<br>Commence food chart if patient unable to communicate oral intake accurately

Patient weight: ____ kg

Referral to Nutrition Department: ☐ Yes ☐ No

Nourishing Diet Commenced: ☐ Yes ☐ No

Food Chart Commenced: ☐ Yes ☐ No

35016(0320)

Falls	< 4 hours No	< 4 hours Yes	ACTIONS	Initial
Is the patient:<br>☐ 65 years and over<br>☐ 45 years and over if Aboriginal and Torres Strait Islander	☐	☐	Complete Falls Risk Assessment & Individualised Interventions<br>Use the Falls Icon	
Has the patient had a fall in the last 12 months?	☐	☐		
Clinically, do you consider the patient at risk of falling?	☐	☐		

MINIMUM INTERVENTIONS

To be implemented for ALL patients as appropriate

- Provide ongoing orientation for patient to bed area, toilet facilities and ward
- Demonstrate the use of call bell, ensure it is within reach and that they can use it effectively
- Ensure frequency used items including mobility aids are within easy reach of patient
- Encourage patient to use their aids such as glasses or hearing aids
- Adjust bed and chair to appropriate height for patient.
- Minimise prolonged bedrest Place IV pole and all other devices/ attachments on exit side of the bed.
- Remove clutter and obstacles from room.
- Provide adequate lighting according to patient's activities/needs
- Encourage patient to take adequate fluids and nutrition.
- Optimise footwear where possible – discourage walking in socks/compression stockings or ill-fitting footwear. Bare feet (if there no infection risk) and non-slip socks are acceptable
- Educate that all inpatients are at increased risk of falling due to injury/illness/medications. e.g. anticoagulation therapy, osteoporosis, deranged blood profiles.

Skin and Pressure Injury	< 4 hours No	< 4 hours Yes	ACTIONS	Initial
Does the patient present with a pressure injury or wound?	☐	☐	If yes to either, complete a full skin assessment AND determine risk using Waterlow Risk Assessment Tool for Pressure Injury	
Does the patient any of the following pressure injury risks?<br>☐ Unable to turn independently<br>☐ Wheelchair bound<br>☐ Multiple co-morbidities<br>☐ Admitted from another location other than home<br>☐ Surgery lasting > 4 hours<br>☐ At nutrition risk (refer to MST)	☐	☐		
Skin Inspection<br>Identify sites for pressure injury and wounds			Contact Tissue Viability Team	
			Complete RiskMan	
			Commence Wound Assessment & Management Form	
			Stage each pressure injury	

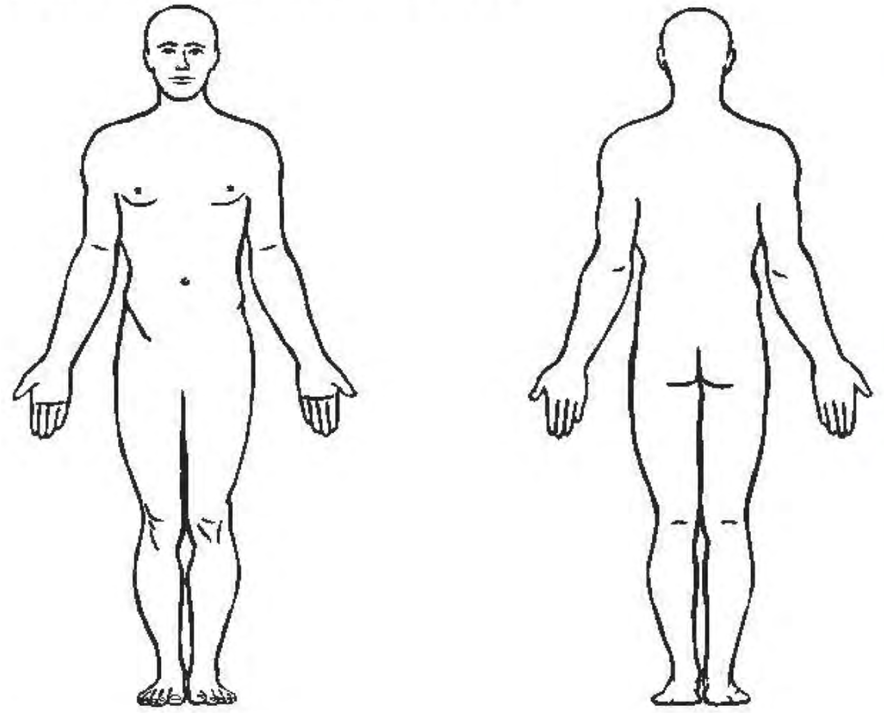

35016(0320)

DO NOT WRITE IN THIS BINDING MARGIN

DO NOT WRITE IN THIS BINDING MARGIN

* 3 5 0 1 6 *

35016(0320)

INTEGRATED PATIENT RISK SCREENING - ADULT

Complete details or affix label

URN: ______________________

Family name: ______________________

Given names: ______________________

DOB: ______________ Sex: ______________

Form to be commenced in ED by Nurse for patients at risk. To Be reviewed and completed on admission to the ward.

Patient presented to: ☐ ED ☐ Direct admission to ward ☐ Other: ______________

Presentation date: _____/_____/_____ Time: _____:_____

☐ Healthcare record has been updated to reflect patient's full name, DOB, address, NOK and GP details (if known)

Reason for admission/brief history: ______________________

Has the patient seen a doctor, or been to the ED for this problem before? ☐ Yes ☐ No

Estimated length of stay or estimated discharge date (EDD): _____/_____/_____

Patient Information provided by: ☐ Patient ☐ NOK ☐ Other: ______________

Initial

Screening questions to be completed within timeframes irrespective of location

Patient Identification	< 4 hours No	< 4 hours Yes	ACTIONS	Initial
Patient has Identification Band in place and 3 identifiers are correct	☐	☐		
Patient identifies as:<br>☐ Aboriginal<br>☐ Torres Strait Islander<br>☐ Both Aboriginal and Torres Strait Islander<br>☐ Prefer not to disclose ☐ Neither	☐	☐	Refer to ALO Service	
Check for:<br>☐ Allergies<br>☐ Adverse drug reactions<br>☐ Other alerts<br>Details:	☐	☐	Document on Alerts Management System, Medication Chart/EMM	
			Apply Red Identification Band if required	
			Phone Nutrition Dept 24/7 if food allergies	

Directives and Legal	< 8 hours No	< 8 hours Yes	ACTIONS	Initial
Does the patient have any of these documents to add to their healthcare record?<br>☐ Advanced Care Plan/Statement of Choices<br>☐ Health Direction ☐ Enduring Power of Attorney<br>☐ Mental Health Act Treatment Order/Advance Care Diriection<br>☐ Guardianship Order and/or Management order<br>☐ Other (e.g. Apprehended Violence Order, Domestic Violence Order)<br>Details:	☐	☐	Ensure a copy of any directive or legal document is included in the clinical record.	
			Ensure alerts are documented in Alerts Management System	

INTEGRATED PATIENT RISK SCREENING - ADULT

35016

Communication	< 4 hours No	Yes	ACTIONS	Initial
First language: ☐ English ☐ Other: ______				
Interpreter required (including Auslan)?	☐	☐	Arrange interpreter	
Does the patient have difficulty talking/understanding?	☐	☐	Refer to Speech Pathology	
Hearing Impairment?<br>If yes: ☐ Hearing aid: ☐ Left ☐ Right ☐ Nil<br>☐ Cochlear implant: ☐ Left ☐ Right ☐ Nil	☐	☐	Auslan interpreter if required	
Vision Impairment?<br>If yes: ☐ Glasses ☐ Contact lenses	☐	☐	Required aids available	

Infection/Infectious Diseases	< 4 hours No	Yes	ACTIONS	Initial
Does the patient have a diagnosed or provisional diagnosis of a notifiable disease?	☐	☐	Medical Officer to notify Public Health as required	
Detail:				
Does the patient have history of:<br>☐ Multi-resistant organism e.g. MRSA, VRE<br>☐ Overseas travel in past 12 months	☐	☐	Implement precautions and seek IPCU advice	
Or symptoms of:<br>☐ Respiratory illness<br>☐ Vomiting and/or diarrhoea	☐	☐		
Has the patient transferred from another hospital or Nursing Home?	☐	☐		
Has the patient had cytotoxic medications in the past 7 days?	☐	☐	Cytotoxic precautions implemented	

Deterioration of Mental State	< 4 hours No	Yes	ACTIONS	Initial
If yes, please indicated which (can indicate more than 1)<br>☐ Suicidal ideation/attempt self-harm<br>☐ Threat of harm to others<br>☐ Psychotic symptoms<br>☐ Withdrawn / uncommunicative<br>☐ Bizarre / disoriented behaviour<br>☐ Significant agitation<br>☐ Unable to rest/risk of misadventure	☐	☐	If yes to any of these, Medical Officer review to consider Mental Health Consultation Liaison Service consultation	

Delirium & Cognitive Impairment	< 4 hours No	Yes	ACTIONS	Initial
Is the patient:<br>☐ 65 years and over<br>☐ 45 years and over if Aboriginal and Torres Strait Islander	☐	☐	If yes, complete Abbreviated Mental Test (AMT) below	
OR have any of the following:<br>☐ Severe illness/risk of dying<br>☐ Hip Fracture<br>☐ Recent surgery<br>☐ Known cognitive impairment /dementia<br>☐ Disruptive behaviour<br>☐ Cognitive concern raised by others / hypoactive state	☐	☐		

35016(0320)

DO NOT WRITE IN THIS BINDING MARGIN

DO NOT WRITE IN THIS BINDING MARGIN

35016(0320)

INTEGRATED PATIENT RISK SCREENING - ADULT

Complete details or affix label

URN: ______

Family name: ______

Given names: ______

DOB: ______ Sex: ______

Abbreviated Mental Test (AMT)		ACTIONS	Initial
**Establish baseline cognition by completing AMT if identified cognition risk.**	**Score 1 for each correct answer**	If score is ≤7, refer to Medical Officer to assess for delirium using **Confusion Assessment Method (CAM) Delirium Screen**	
1. How old are you?			
2. What is the time? (nearest hour)<br>Give the patient an address and ask them to repeat it at the end of the test.<br>e.g. 42 Smith Street, Kingston			
3. What year is it?			
4. What is the name of this place?			
5. Can the patient recognise two relevant persons?			
6. What is your date of birth?			
7. When did the second World War start (1939)?			
8. Who is the current Prime Minister?			
9. Count backwards from 20 to 1.			
10. Can you remember the address I gave you?			
**TOTAL SCORE**			

Medication	< 4 hours No	Yes	ACTIONS	Initial
Does the patient take any regular medication?	☐	☐	Advise Medical Officer	
Did they bring in their own medication?	☐	☐	Document where medication is stored	
	**< 8 Hours**		**ACTIONS**	**Initial**
Does the patient use more than 5 medications?	☐	☐	Refer to JMO or Ward Pharmacist for medication review	
Does the patient use high risk medications such as:<br>☐ Insulin<br>☐ Opiod analgesics ☐ Benzodiazepines<br>☐ Chemotherapy<br>☐ Warfarin and other oral anticoagulants<br>☐ Clozapine<br>☐ Immunosuppressants or transplant medication	☐	☐		

Function *(tick if assistance is required)*

On presentation (< 4 hours)	Usual level of function (< 8 hours)	ACTIONS	Initial
☐ Nil assistance required<br>☐ Eating ☐ Toileting<br>☐ Oral Hygiene ☐ Bathing<br>☐ Dressing<br>☐ Transfers<br>☐ Mobility<br>☐ Mobility aid ______<br>☐ Independent with aid<br>☐ Supervision<br>☐ Assist x1 ☐ Assist x2	☐ Nil assistance required<br>☐ Eating ☐ Toileting<br>☐ Oral Hygiene ☐ Bathing<br>☐ Dressing<br>☐ Transfers<br>☐ Mobility<br>☐ Mobility aid ______<br>☐ Independent with aid<br>☐ Supervision<br>☐ Assist x1 ☐ Assist x2	Encourage support with ADLs<br>Supervise / assist mobility, transfers<br>Source required mobility aid<br>If increased assistance required for mobility and ADL<br>Refer to Physio & OT for assessment	

* 3 5 0 1 7 *

DO NOT WRITE IN THIS BINDING MARGIN

INTEGRATED PATIENT RISK ASSESSMENTS - ADULT

Complete details or affix label

URN: ______________________

Family name: ______________________

Given names: ______________________

DOB: ______________ Sex: ______________

Pressure Injury Risk Assessment (Waterlow[2])

Circle applicable score. Add total score. Several scores may be selected in some categories

Sex and Age		Skin Type and Visual Areas		Continence		Tissue Malnutrition	
Male	1	Healthy	0	Complete/Catheterised	0	e.g.	
Female	2	Tissue paper	1	Occasionally incontinent	1	Smoking	1
14-49	1	Dry	1	Catheter/incontinent of faeces	2	Anaemia	2
50-64	2	Oedematous	1	Doubly incontinent	3	Peripheral Vascular disease	5
65-74	3	Clammy	1			Cardiac Failure	5
75-80	4	Discoloured	2			Terminal Cachexia	8
80+	5	Broken	3				

Mobility		Neurological Deficit		Appetite		Build/Weight for Height	
Fully	0	(e.g. Diabetes, MS, CVA, Motor/sensory paraplegia)		Average	0	Average	0
Restless/fidgety	1			Poor	1	Above average	1
Apathetic	2			NG tube/fluids only	2	Obese	2
Restricted	3	Moderate	4	NBM/anorexia	3	Below average	3
Inert/traction	4	Moderate-severe	5	**Major Surgery/Trauma**		**Medication**	
Chair-bound	5	Severe	6	Orthopaedic – below waist, spinal	5	Cytotoxics	
				On table > 2 hrs (within last 48 hrs)	5	High Dose Steroids	
						Anti-inflammatory	4

10+ At Risk	15+ High Risk	20+ Very High Risk	Risk Score	
Implement prevention strategies within 2 hours	Implement prevention strategies within 2 hours	Implement prevention strategies within 30 mins		

[2] Pressure Injury Adapted from Judy Waterlow Risk Assessment Tool

☐ **Pressure Injury Information Provided**

If AT RISK for pressure injury, document management and interventions in Comprehensive Care Plan

Signature ______________ Print name ______________ Designation ______________ Date/Time ______________

Falls Risk Assessment and Individualised Interventions

If patient has been admitted or transferred from another ward; or had a fall; or medically deteriorated or improved? If YES to any then screen is indicated

MOBILITY and FUNCTIONAL ABILITY RISKS				Initial if appropriate
	**No**	**Yes**	**Action/Intervention**	
Does the patient require assistance with mobility or transfers?	☐	☐	Educate patient on the level of assistance required (including aids) and or need to call for and wait for assistance	
Does the patient have poor coordination, balance, gait or uncorrected visual impairment?	☐	☐	Refer to Physiotherapist for comprehensive mobility assessment	
			Document and provide mobility aids and assistance required	
Is the patient unsteady, disorganised or require assistance when attending to ADLs?	☐	☐	Refer to Occupational Therapist for functional assessment.	
**MEDICATIONS/MEDICAL CONDITION RISKS** Some medications are associated with falls				Initial if appropriate
Has the patient been prescribed psychoactive medications e.g. benzodiazepines, antipsychotics, antidepressants?	☐	☐	Liaise with Medical Officer or Pharmacist for review of medication associated with falls	

35017(0320)

INTEGRATED PATIENT RISK ASSESSMENTS - ADULT

35017

Has the patient been prescribed new or old medication that may affect their blood pressure?	☐	☐	If reported dizziness, check lying/standing blood pressure. If postural drop >20mmHg systolic or >10mmHg diastolic present, discuss care plan with MO.	
Does the patient take more than 5 medications of any sort?	☐	☐		
Does the patient report dizziness or presented following a fall/collapse?	☐	☐	Educate patient to stand up slowly and wait until dizziness resolves before mobilising	
**COGNITIVE STATE RISKS**				**Initial if appropriate**
In selective patient groups, AMT was completed in ***Integrated Patient Risk Screening - Adult*** form	☐	☐	If result is abnormal, (e.g. AMT < 7) refer to MO for prompt review	
	☐	☐	Remain in attendance at all times when patient is toileting or showering as this is high risk activity for patient.	
	☐	☐	If agitated, commence behaviour observation chart to assist behaviour management plan	
	☐	☐	Avoid use of bed rails due to climbing/ entrapment risk and consider high-low bed	
	☐	☐	Reorientate patient and ask family to assist in orientating and settling patient.	
	☐	☐	Increase frequency of patient check to proactively attend to patient needs.	
**CONTINENCE /ELIMINATION RISKS**				**Initial if appropriate**
Does the patient require assistance with toileting?	☐	☐	Monitor/record toileting needs to check frequency, retention or constipation.	
Does the patient have constipation, urinary or faecal frequency/urgency or nocturia?	☐	☐	Review toileting needs with patient daily including frequency, patient requirement for continence/toileting aids and assistance required to reach toilet facilities	
			Complete urinalysis. If abnormal, discuss with MO if MSU indicated	

Signature	Print name	Designation	Date/Time

PATIENT REQUIRES INTERVENTIONS OTHER THAN ABOVE – complete section below

Interventions can be added by any member of the MDT when discussed with the CNC – e.g. Nurse, Allied Health, Medical Officer, Pharmacist

Name, designation & signature	Date	Intervention *e.g. bed alarms*	Date actioned & by whom	Date ceased and by whom

DO NOT WRITE IN THIS BINDING MARGIN

35017(0320)

DO NOT WRITE IN THIS BINDING MARGIN

* 6 5 0 1 8 *

Complete details or affix label

URN: ______________________

Family name: ______________________

Given names: ______________________

DOB: ______________ Sex: __________

COMPREHENSIVE CARE PLAN - ADULT

Day:<br>Date: ____/____/____	Individual Goals of Care	Shift	Initial	Variance/comments
**OBSERVATIONS AND FREQUENCY**<br>Vital signs, O_2 requirement<br>Weight, BGL<br>Other		AM		
		PM		
		Night		
**INFECTION PREVENTION**<br>precautions<br>IDC, intravascular management<br>Wound plan, other		AM		
		PM		
		Night		
**NUTRITION, HYDRATION**<br>Diet type and restrictions<br>Assistance required<br>Oral, enteral – NG/Peg/other<br>Fluid balance, IVT		AM		
		PM		
		Night		
**ELIMINATION**<br>IDC/SPC, continence aids, stoma<br>Self-caring/assistance		AM		
		PM		
		Night		
**FUNCTION /HYGIENE**<br>Transfers and mobility<br>Oral hygiene/bathing/dressing<br>Equipment and assistance required		AM		
		PM		
		Night		
**COGNITION/BEHAVIOUR**<br>Confusion/memory<br>Impulsivity, poor initiation, other<br>Communication, aids required		AM		
		PM		
		Night		
**FALLS**<br>Equipment and assistance required<br>Minimum interventions only<br>Min. and individualised interventions		AM		
		PM		
		Night		
**PRESSURE INJURY**<br>Waterlow Risk Score (assess daily)<br>Skin assessment<br>Interventions		AM		
		PM		
		Night		
**SOCIAL, WELLBEING**<br>Cultural needs<br>Disability, other		AM		
		PM		
		Night		
**REFERRALS /PATHWAYS**<br>Specialist, multidisciplinary team<br>Discharge planning<br>Other care plans/pathways		AM		
		PM		
		Night		

Signature ______________ Print name ______________ Designation ______________ Date/Time ______________

Patient/Carer involved in the development of the patient's care plan

Signature: ______________________ Print name: ______________________

65018(0320)

COMPREHENSIVE CARE PLAN - ADULT

65018

Complete details or affix label

URN: ______

Family name: ______

Given names: ______

DOB: ______ Sex: ______

COMPREHENSIVE CARE PLAN - ADULT

Day: Date: ____/____/____	Individual Goals of Care	Shift	Initial	Variance/comments
**OBSERVATIONS AND FREQUENCY**<br>Vital signs, O_2 requirement<br>Weight, BGL<br>Other		AM		
		PM		
		Night		
**INFECTION PREVENTION**<br>precautions<br>IDC, intravascular management<br>Wound plan, other		AM		
		PM		
		Night		
**NUTRITION, HYDRATION**<br>Diet type and restrictions<br>Assistance required<br>Oral, enteral – NG/Peg/other<br>Fluid balance, IVT		AM		
		PM		
		Night		
**ELIMINATION**<br>IDC/SPC, continence aids, stoma<br>Self-caring/assistance		AM		
		PM		
		Night		
**FUNCTION /HYGIENE**<br>Transfers and mobility<br>Oral hygiene/bathing/dressing<br>Equipment and assistance required		AM		
		PM		
		Night		
**COGNITION/BEHAVIOUR**<br>Confusion/memory<br>Impulsivity, poor initiation, other<br>Communication, aids required		AM		
		PM		
		Night		
**FALLS**<br>Equipment and assistance required<br>Minimum interventions only<br>Min. and individualised interventions		AM		
		PM		
		Night		
**PRESSURE INJURY**<br>Waterlow Risk Score (assess daily)<br>Skin assessment<br>Interventions		AM		
		PM		
		Night		
**SOCIAL, WELLBEING**<br>Cultural needs<br>Disability, other		AM		
		PM		
		Night		
**REFERRALS /PATHWAYS**<br>Specialist, multidisciplinary team<br>Discharge planning<br>Other care plans/pathways		AM		
		PM		
		Night		

Signature ______ Print name ______ Designation ______ Date/Time ______

Patient/Carer involved in the development of the patient's care plan

Signature: ______ Print name: ______

DO NOT WRITE IN THIS BINDING MARGIN

65018(0320)
