# Supplementary material for: ‘Paper care not patient care’: Nurse and patient experiences of comprehensive risk assessment and care plan documentation in hospital
Source: J Clin Nurs. 2022 Mar 29;32(3-4):523–38. doi: 10.1111/jocn.16291 (PMC10084263; doi:10.1111/jocn.16291)
Supplement: Supplementary file 4 — Supplementary Material [file JOCN-32-523-s001.pdf]

RISK SCREENING - ADULT

Complete details or affix label

URN: ______________________

Family name: ______________________

Given names: ______________________

DOB: ______________ Sex: ______________

Presenting Problem

Relevant Patient Medical/Surgical History and Pre-Hospital Interventions

Alert/Allergy

Alert/Allergy	Yes	No
Patient has known alerts/allergies?<br>Details:	☐	☐
If Yes: ☐ Activate Alert Management System ☐ Alert ID band		

PART A: Primary Assessment *(complete on presentation)*

A - AIRWAY	B - BREATHING	C - COLOUR	C - CIRCULATION	D - CONSCIOUS STATE	E - SKIN
☐ Patent	☐ Spontaneous	☐ Natural	☐ HR Regular	☐ Alert	☐ Warm
☐ Compromised	☐ Trach Midline	☐ Pale	☐ HR Irregular	☐ Responds to Voice	☐ Hot
☐ C-spine immob	☐ Air Entry OK	☐ Flushed	☐ HR Slow	☐ Responds to Pain	☐ Cool
	☐ WOB Increased	☐ Mottled	☐ HR Fast	☐ Unresponsive	☐ Clammy
	☐ Stridor/Wheeze	☐ Cyanotic	☐ Cap refill <3 sec	☐ Lethargic	☐ Cold
	☐ Cough	☐ Jaundiced		☐ Agitated	☐ Rash
	☐ Grunting	☐ Grey		☐ PERTL	☐ Bruises
					☐ Broken

Police Bloods ☐ Attended ☐ Refused Time: ______:______ ☐ Sticker Tube No. : ______________

Patient Identification

Patient Identification	Yes	No
Patient is positively identified and has an arm band in place (red ID band for alert). Patient confirms.	☐	☐
Correct ID and spelling? (or if unable, second staff member to check)	☐	☐
Does the patient identify as: ☐ Aboriginal ☐ Torres Strait Islander ☐ Aboriginal & Torres Strait Islander ☐ Prefer not to disclose	☐	☐
If Yes: ☐ Activate Alert Management System ☐ Refer to ALO ☐ Include in Care Plan		

Communication

Communication	Yes	No
English IS NOT the patient's primary language?	☐	☐
Does the patient have hearing or speech difficulties?	☐	☐
Does the patient require an interpreter?	☐	☐
If Yes: ☐ Activate Alert Management System ☐ Include in Care Plan ☐ Interpreter offered ☐ Interpreter provided		

Unapproved DRAFT Not for Clinical Use

DO NOT WRITE IN THIS BINDING MARGIN

Patient Belongings		Yes	No
Taken by relative: Name:	Contact No:	☐	☐
Clothing cut off	☐ Discarded with permission	☐	☐
Forensic collection		☐	☐
Valuables in Safe Receipt No:		☐	☐
Valuables remain with patient *(description)*		☐	☐

NOK/Support Person Contact Details

NOK Name: ______________________ Relationship: ______________

Phone: ______________________ ☐ Contacted ☐ Present

Support person name: ______________________ Relationship: ______________

Phone: ______________________ ☐ Contacted ☐ Present

Completing Clinician

Signature Print name Designation Date Time

Interventions

Date	Time	Intervention	Gauge	Site
		IVC	Gauge	Site
		IVC	Gauge	Site
		CVC/PICC	Gauge	Site ☐ X-ray check
		I/O / Other		
		NGT/OGT		☐ Position confirmed
		IDC		☐ Urethral ☐ Suprapubic
		Other		

Unapproved DRAFT Not for Clinical Use

PART B: Comprehensive Screening

1. Directives and Legal	Yes	No
Does the patient have any of the following to add to their health record?<br>☐ Advance Care Plan/Statement of Choices ☐ Health Direction ☐ Enduring Power of Attorney<br>☐ Other Details:	☐	☐
If Yes: ☐ Copy to be included in the Clinical Record ☐ Activate Alert Management System		

Signature Print name Designation Date Time

2.1 Sepsis *(all patients 16 years and over) (to be completed within 4 hours of presentation)*	Yes	No
Does the patient look unwell?	☐	☐
Does the patient have recent or current fever?	☐	☐
Does the patient have hypothermia (<35.5°C)?	☐	☐
Does the patient have a suspected infection?	☐	☐
You suspect the patient may have sepsis?	☐	☐
Signs of clinical deterioration (MEWS $\geq$ 4)?	☐	☐
If Yes to ANY: ☐ ED Sepsis pathway commenced ☐ MO notified ☐ Mews escalation activated		

Signature Print name Designation Date Time

DO NOT WRITE IN THIS BINDING MARGIN

Complete details or affix label

URN: ____________________

Family name: ____________________

Given names: ____________________

DOB: ____________________ Sex: ____________________

RISK SCREENING - ADULT

DO NOT WRITE IN THIS BINDING MARGIN

2.2 Infection and disease prevention	Yes	No
Does the patient have a diagnosed or provisional diagnosis of a notifiable disease?<br>Details:	☐	☐
Have ANY samples been taken for testing?<br>Details:	☐	☐
Does the patient have a history of:<br>☐ Multi resistant organisms e.g. MRSA, VRE Specify: ____________	☐	☐
Recent overseas/interstate travel in the last 12 months or current symptoms of:<br>☐ Respiratory illness ☐ Vomiting and/or diarrhoea	☐	☐
Has the patient transferred from another hospital, nursing home or other residential care facility?	☐	☐
Has the patient had cytotoxic medication in the last 7 days?	☐	☐
If Yes to ANY: ☐ Implement appropriate precautions ☐ Surveillance swabs taken		

Signature Print name Designation Date Time

3. Medication Questions	Yes	No
Does the patient take more than 5 medications?	☐	☐
If Yes: ☐ Request Pharmacy review ☐ Include on Care Plan		

Signature Print name Designation Date Time

4. Skin and Pressure Injury *(to be completed within 4 hours of presentation)*	Yes	No
Does the patient present with a pressure injury or wound?	☐	☐
Does the patient have any of the following pressure injury risks?<br>☐ Unable to move independently<br>☐ Wheelchair bound<br>☐ Multiple co-morbidities<br>☐ Admitted from another location other than home<br>☐ At nutrition risk (refer to MST)	☐	☐
If Yes: ☐ Complete skin assessment and Waterlow Risk Assessment Tool ☐ Activate Alert Management System ☐ Include in Care Plan		

Unapproved DRAFT
Not for Clinical Use

Skin Inspection *(Identify sites for pressure injury and wounds)*

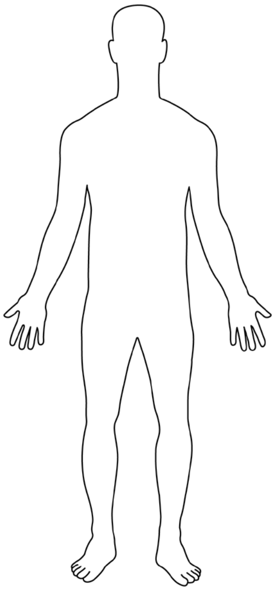

Key	
#	Fracture
A	Abrasion
B	Burn
BR	Bruising
C#	Compond #
L	Laceration
LU	Leg Ulcer
P	Pressure Injury
S	Swelling
ST	Skin Tear
T	Tenderness

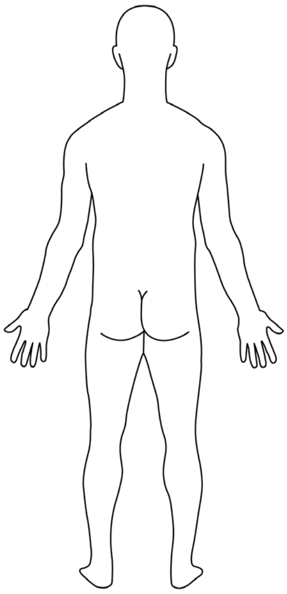

Signature Print name Designation Date Time

5. Delirium, Cognitive Impairment *(to be completed within 4 hours of presentation)*

	Yes	No
Does the patient have any of the following:<br>☐ Severe illness/risk of dying ☐ Hip fracture ☐ Known cognitive impairment/dementia<br>☐ Recent surgery ☐ Disruptive behaviour<br>☐ Cognitive concern raised by others/hypoactive/hyperactive/mixed state<br>☐ Recent onset of confusion, anxiety or hallucinations	☐	☐

If Yes: ☐ Attend 4AT screening ☐ If 4AT score>1 Activate Alert Management System
☐ Request CAM and pathology screening

Signature Print name Designation Date Time

6. Falls *(to be completed within 4 hours of presentation)*

	Yes	No
Is the patient 65 years of older (45 years and older if Aboriginal, Torres Strait Islander)	☐	☐
Has the patient had a fall in the last 6-12 months?	☐	☐
Clinically do you consider the patient at risk of falling?	☐	☐

If Yes to any: ☐ Completed falls assessment within 4 ☐ Activate Alert Management System
☐ hours Reassess daily as per Care Plan

Signature Print name Designation Date Time

7. Mental State

	Yes	No
Have any of the following signs of deterioration in mental state been reported or observed?<br>☐ Verbal commands to do harm to self or others ☐ Suicidal ideation<br>☐ Attempt at self harm ☐ Threat of harm to others<br>☐ Withdrawn/uncommunicative ☐ Agitation<br>☐ Restlessness ☐ Ambivalence about treatment<br>☐ Physical/verbal aggression ☐ Mood disturbance *(depression, elevated or irritable mood)*<br>☐ Psychotic symptoms *(hallucinations, delusions, paranoid ideas)*	☐	☐

If Yes: ☐ Consider Mental Health Consultation ☐ Activate Alert Management System
☐ Liaison Include in Care Plan

Signature Print name Designation Date Time

Unapproved DRAFT Not for Clinical Use

DO NOT WRITE IN THIS BINDING MARGIN

Complete details or affix label

URN: ______________________

Family name: ______________________

Given names: ______________________

DOB: ______________ Sex: ______________

RISK SCREENING - ADULT

DO NOT WRITE IN THIS BINDING MARGIN

Unapproved DRAFT
Not for Clinical Use

8. Nutrition

8. Nutrition	Yes	No
Do you have trouble swallowing your food, drinks or tablets?	☐	☐

If Yes: ☐ Activate Alert Management System ☐ Include in Care Plan ☐ Refer to Speech Pathology

Malnutrition Screening Tool

Date: _____/_____/_____	Response	Score
1. Has the patient lost weight recently without trying?	☐ No	0
	☐ Unsure	2
	☐ Yes 1-5kg	1
	☐ Yes 6-10kg	2
	☐ Yes 11-15kg	3
	☐ Yes > 15kg	4
	☐ Yes - unsure	2
2. Has the patient been eating poorly because of a decreased appetite?	☐ No	0
	☐ Yes	1
Patients weight: ________ kg	**Total**	

MST score	Malnutrition Risk Response
0-1	1. Continue current diet<br>2. Rescreen weekly
2	1. Call Nutrition Department and request nourishing diet<br>2. Re-screen weekly<br>3. Consider starting food chart
3-5	1. Call Nutrition Department and request nourishing diet and dietitian assessment<br>2. Commence food chart if patient unable to communicate oral intake accurately

Refer to Nutrition Department? ☐ Yes ☐ No

Signature | Print name | Designation | Date | Time

9. ADL Function

9. ADL Function	Yes	No
Does the patient require assistance with:	☐	☐

☐ Eating ☐ Toileting ☐ Oral hygiene ☐ Bathing ☐ Dressing ☐ Transfers

☐ Mobility Mobility aid: ____________ ☐ Independent with aid ☐ Supervision

☐ Assist x1 ☐ Assist x2 ☐ Prosthesis Prosthesis type: ______________________

If Yes: ☐ Activate Alert Management System ☐ Include in Care Plan ☐ Refer OT/PT

Signature | Print name | Designation | Date | Time

10. End of Life

10. End of Life	Yes	No
Is the patient 65 years of older (45 years and older if Aboriginal, Torres Strait Islander)	☐	☐
**AND** does the patient present with **2 or more** of the following:<br>☐ Poor or deteriorating health<br>☐ Previous unplanned hospital admission within the last 12 months<br>☐ Life limiting illness or disability<br>☐ Family express concern about quality of life	☐	☐

If Yes to **BOTH**: ☐ Consider referral to MO to conduct End of Life Screening

	Yes	No
Would you be surprised if this person died in the next 30 days?	☐	☐

If No: ☐ Refer to Medical Officer to conduct End of Life Screening Tool

Signature | Print name | Designation | Date | Time

11. Patient/Carer Consultation Declaration	Yes	No
Patient and/or support person have been consulted during the risk screening process and have been informed of the need for any further assessments and referrals that arise from the screening?	☐	☐

Signature Print name Designation Date Time

Patient Disposition

**Ward Transfer - Use ISBAR** *(Introduction, situation, background, assessment, recommendation)*	Yes	No	N/A	**Discharge Home**	Yes	No	N/A
Phone Handover	☐	☐		IVC removed	☐	☐	☐
Medication Chart	☐	☐	☐	DLN review	☐	☐	☐
IVF Chart	☐	☐	☐	Discharge summary	☐		
MEWS / PEWS < 4	☐	☐		Belongings provided	☐	☐	
If no, Management Plan documented	☐	☐		Valuables	☐	☐	☐
If risks identified in comprehensive assessment handed over on transfer	☐			Dentures	☐	☐	☐
				Glasses	☐	☐	☐

Advise during handover if Alerts have not been placed on Alert Management System or documented in Care Plan.

Sign for handover

Signature Print name Ward Nurse Date Time

Discharging/Transferring Clinician

Signature Print name Designation Date Time

Unapproved DRAFT
Not for Clinical Use

Date / Time	**Pre-Admission Progress Notes only:** All entries to be dated, timed, signed, name printed and designation indicated

DO NOT WRITE IN THIS BINDING MARGIN

DO NOT WRITE IN THIS BINDING MARGIN

RISK SCREENING - ADULT

Complete details or affix label

URN: ______________________________

Family name: ______________________________

Given names: ______________________________

DOB: ______________ Sex: ______________

Date / Time	**Pre-Admission Progress Notes only:** All entries to be dated, timed, signed, name printed and designation indicated

Unapproved DRAFT
Not for Clinical Use

This page has been left blank intentionally
DO NOT WRITE ON THIS PAGE
Anything written on this page will not be saved into the Clinical Record

Unapproved DRAFT
Not for Clinical Use

Complete details or affix label

URN: ____________________

Family name: ____________________

Given names: ____________________

DOB: ____________________ Sex: ____________

CARE PLAN - ADULT

Complete appropriate Care Plan section for each shift

Reason for admission: ____________________ **Date:** __________ **Number of Days admitted:** ______ **EDD:** __________ **Ward:** ______

**Handover Notes**	AM	PM	ND
**Feedback from MDT meeting:** ____________________<br>*Use this section to highlight points to be noted in handover e.g. expected tests, MDT outcomes.*<br>*Use ISBAR to handover*			

	Complete on Morning Shift or Shift of Admission	Comment/Variance AM	Comment/Variance PM	Comment/Variance NIGHT	Ceased<br>Initial
**Observations and Frequency**<br>**Issue/Problem:** ______<br>**Goal:** ______	**Vital signs:** Frequency: ______ **O_2** requirements: ______ ☐ **BGL:** Frequency: ______<br>**Weight:** Frequency: ______ **Date Due:** ______ ☐ **Weight noted on chart**<br>☐ **Other observations** (specify): ______ ☐ Mental Health check	(note changes)	(note changes)	(note changes)	
**Input** **Nutrition:**<br>**Issue/Problem:** ______<br>**Goal:** ______<br>*How long has your patient been fasting?*<br>**Intravenous:**<br>*Does your patient need IV access? Can it be removed?*	☐ Oral Specify diet, including restrictions: ______<br>Food assistance: ☐ Nil ☐ Full feed ☐ Set up ☐ Food chart<br>☐ NBM NBM reason: ______ No. days NBM: ______ ☐ TPN<br>☐ Enteral *(circle route)* NG / PEG / Other: ______ Feed type: ______<br>Line type/site: ______ Insertion date: ______ Dressing/resite due: ______ Cap due: ______<br>Line type/site: ______ Insertion date: ______ Dressing/resite due: ______ Cap due: ______<br>Line type/site: ______ Insertion date: ______ Dressing/resite due: ______ Cap due: ______	(note new lines and location)	(note new lines and location)	(note new lines and location)	
**Output**<br>**Issue/Problem:** ______<br>**Goal:** ______<br>**Fluid Balance Chart Required?** ☐ **Yes** ☐ **No**	**Urine:** ☐ Self Caring ☐ IDC/SPC Date of insertion: ______ ☐ Stoma<br>☐ Assist/Pan/Urinal ☐ Incontinent Abdomen measurement for continence aid size (cm): ______<br>**Drains:** Specify site/s and special orders: ______<br>**NG:** ☐ Free drainage with ______ hourly aspiration Special orders: ______<br>**Bowels:** ☐ Self Caring ☐ Assist/Pan ☐ Incontinent ☐ Stoma ☐ Stool Chart	**Fluid Balance Chart**<br>☐ Yes ☐ No	**Fluid Balance Chart**<br>☐ Yes ☐ No	**Fluid Balance Chart**<br>☐ Yes ☐ No	
**Falls**<br>**Falls Risk?** ☐ **Yes** ☐ **No**<br>**Reassess if patient has** transferred ward, had a fall, medically deteriorated/improved, post-surgery, change in condition<br>**Goal:** ______<br>☐ Education provided	**Do the following for ALL patients 'AT RISK' of falls:**<br>☐ 'Falls risk' sign above bed ☐ Conduct bed rail assessment ☐ Call bell within reach<br>**Interventions in place from Falls Risk Assessment:**<br>☐ Refer to Allied Health for further assessment ☐ Mobility aid provided and within reach<br>☐ Medical/Pharmacist medication review ☐ Postural Hypotension assessment<br>☐ Supervision for toileting and showering ☐ Regular rounding<br>☐ Orientate patient to bed area, bathroom, and ward ☐ Remove clutter and obstacles from room	(note changes and reassess if required)	(note changes and reassess if required)	(note changes and reassess if required)	
**Pressure Injury**<br>**Issue/Problem:** ______<br>**Goal:** ______<br>**PI present on admission?** ☐ **Yes** ☐ **No**<br>**Waterlow Risk Score** (assess daily & if condition changes): ______<br>☐ Tick if PI education provided	**Assess:** Skin Intact ☐ Yes ☐ No Pressure Injury site/s: ______<br>☐ Stage 1 ☐ Stage 2 ☐ Stage 3 ☐ Stage 4 ☐ Unstagable ☐ Suspected Deep Tissue Injury<br>**Interventions:** ☐ 2 hourly turns ☐ 4 hourly turns ☐ Self Caring<br>Heels offloaded / suspension device used ☐ Yes ☐ No ☐ Active air cushion ☐ Active air mattress<br>Preventative foam sacral/heel dressing ☐ Yes ☐ No Specify where: ______<br>☐ Moisturise skin daily ☐ Nutrition Review ☐ Refer to Tissue Viability Unit<br>***Use Wound Care section below for any dressings***	(note changes and reassess if required)	(note changes and reassess if required)	(note changes and reassess if required)	
**Wound Care**<br>**Issue/Problem:** ______<br>**Goal:** ______	**No. of wounds:** ______ **Locations/s:** ______<br>☐ Referred to tissue viability unit Date: ______ ☐ Wound assessment and management form	(note changes)	(note changes)	(note changes)	
**Mobility/Manual Handling**	**Lifting aid required:** ______ **Mobility aid required:** ______<br>**Staff Assist:** ☐ 1 nurse ☐ 2 nurses ☐ Self Caring ☐ Confined to bed	(note changes)	(note changes)	(note changes)	
**ADLs**<br>**Issue/Problem:** ______<br>**Goal:** ______	**Hygiene:** ☐ Self Caring ☐ Shower ☐ Assistance required: ______<br>Other/notes/special cleanser required: ______<br>______ **Mouth Care:** ☐ Self Caring ☐ Assist	☐ Bedside equipment check complete	☐ Bedside equipment check complete	☐ Bedside equipment check complete	

Continue Care Plan on page 2

Unapproved DRAFT
Not for Clinical Use

DO NOT WRITE IN THIS BINDING MARGIN

CARE PLAN - ADULT

65004

trim when printed on A3

trim when printed on A3

	Assessment and Diagnosis	Planning	Implementation	Evaluation AM	Evaluation PM	Evaluation NIGHT	Ceased
	**Issue / Problem**	**Agreed Goal of Care**	**Action**	**Comment / Variance**	**Comment / Variance**	**Comment / Variance**	**Initial**
**Personal Goals**	**What's important for you today?** *Ask the patient what it is they would like to happen today*						
**Condition Specific Goals**	Pain / discomfort due to:	Pain to be controlled					
**Functional Goals**	Communication and health literacy.<br>Potential for patient not understanding due to: *Assess for communication barriers, e.g. CALD, disability, NESB*	Effective communication with patient. Ensuring the patient has good understanding.	*Include in Alert Management System*				
	Social/cultural - *specific religious cultural needs*						
	Discharge planning:<br>*Is proactive and commences on day of admission.*<br>*Review EDD daily.*<br>*Discuss patient needs when going home.*<br>*Is discharge transport and accommodation appropriate?*<br>*Refer to DLN and other services if appropriate.*						

☐ Patient and/or ☐ support person (Name: ______________________________) have been involved in the formulation of this care plan.

Shift completing care plan ☐ **AM** ☐ **PM** ☐ **ND** ☐ Bedside equipment check complete

Signature ______ Print name ______ Designation ______ Date ______ Time: ______

Evaluation AM	Evaluation PM	Evaluation NIGHT
**Signature:** ______	**Signature:** ______	**Signature:** ______
**Print name:** ______	**Print name:** ______	**Print name:** ______
**Designation:** ______	**Designation:** ______	**Designation:** ______
**Date:** ______ **Time:** ______	**Date:** ______ **Time:** ______	**Date:** ______ **Time:** ______

DO NOT WRITE IN THIS BINDING MARGIN

Unapproved DRAFT
Not for Clinical Use
